# Emergence of β1 integrin-deficient breast tumours from dormancy involves both inactivation of p53 and generation of a permissive tumour microenvironment

**DOI:** 10.1038/s41388-021-02107-7

**Published:** 2021-11-15

**Authors:** Tung Bui, Yu Gu, Frédéric Ancot, Virginie Sanguin-Gendreau, Dongmei Zuo, William J. Muller

**Affiliations:** 1 Rosalind and Morris Goodman Cancer Institute, McGill University, Montreal, H3A 1A3 Canada; 2 Department of Biochemistry, McGill University, Montreal, H3G 1Y6 Canada; 3 Faculty of Medicine, McGill University, Montreal, H3G 2M1 Canada

**Keywords:** Breast cancer, Cell growth, Extracellular signalling molecules, Senescence

## Abstract

The molecular and cellular mechanisms underlying mammary tumour dormancy and cancer recurrence are unclear and remain to be elucidated. Here, we report that mammary epithelial-specific disruption of β1 integrin in a murine model of Luminal B human breast cancer drastically impairs tumour growth with proliferation block, apoptosis induction and cellular senescence. β1 integrin-deficient dormant lesions show activation of the tumour suppressor p53, and tumours that circumvent dormancy possess p53 mutation analogous to those in human disease. We further demonstrate that mammary epithelial deletion of p53 in β1 integrin-deficient mice fully rescues tumour dormancy and bypasses cellular senescence. Additionally, recurrent β1 integrin-deficient tumours exhibit fibrosis with increased cancer-associated fibroblast infiltration and extracellular matrix deposition, absent in fast-growing β1 integrin/p53-deficient lesions. Taken together, these observations argue that β1 integrin modulates p53-dependent cellular senescence resulting in tumour dormancy and that pro-tumourigenic stromal cues and intrinsic genetic mutation are required for dormancy exit.

## Introduction

Tumour dormancy is a major hurdle in the clinical management of breast cancer due to its detrimental roles in therapy resistance and lethal disease recurrence. Dormancy can occur at both cellular and tumour levels, referred to as cellular dormancy and tumour mass dormancy, respectively [1]. Dormant cancer cells generally enter a survival state defined by temporary cell cycle arrest before reactivation and proliferation. In tumour mass dormancy, the constituent cancer cells are kept in check by an equilibrium of cell proliferation and apoptosis, resulting in stagnation of overall tumour growth. Recent evidence also indicates a role of cellular senescence in dormancy and malignant progression [2]. Current literature highlights tumour mass dormancy as a dynamic and multi-stage process controlled by angiogenic and/or immunologic equilibrium and that dormant cancer cells’ interaction with the stromal components can shift the balance in favour of malignant progression.

Emerging studies of breast cancer have demonstrated the importance of the extracellular matrix (ECM) and integrin receptors not only in promoting breast malignancy but also in regulating the maintenance of and the exit from dormancy [3, 4]. Integrin receptors comprise heterodimeric α and β subunits that facilitate cell adhesion to ECM and regulate diverse signalling and cellular processes including cell cycle regulation, survival, and motility [5]. In breast cancer, progressive remodelling of the ECM environment and elevated integrin activity promote malignant transformation and metastatic progression [5]. Specifically, collagen cross-linking and ECM stiffening enhance focal adhesion formation/integrin signalling and provide survival signals for cancer cells [6]. Consistent with this observation, mammary epithelial specific disruption of integrin receptors like β1 integrin or their associated downstream signalling proteins (c-Src, ILK and FAK) in Genetically Engineered Mouse Model (GEMM) of human breast cancer results in impairment of tumour progression [7–10]. Particularly, mammary epithelial deletion of β1 integrin in PyV mT GEMM of Luminal B breast drastically impairs tumourigenesis and exhibits many of hallmarks of tumour dormancy [7]. Together, these observations establish a key role for integrins in incorporating environmental cues with oncogenic signalling to permit breast cancer progression.

However, one key caveat of this β1 integrin-deficient transgenic system is that the stochastic expression of MMTV/Cre transgene in the PyV mT model gave rise to ‘escapee’ tumours retaining β1 integrin expression due to the lack of Cre recombinase expression [7]. To circumvent this issue, we recently developed a Doxycycline-inducible GEMM that co-expresses both PyV mT and Cre recombinase on a polycistronic mRNA [11]. Upon Doxycycline induction, mammary epithelial cells carrying the mammary specific rtTA transgene will activate the coupled expression of PyV mT and Cre recombinase for excision of β1 integrin alleles (*Itgb1*). Consistent with our previous results, mammary-specific ablation of β1 integrin significantly inhibited tumour initiation and progression to invasive disease, resulting in the formation of dormant lesions with slow tumour growth rate correlated with increased cell cycle arrest, apoptosis and cellular senescence. We further showed activation of tumour suppressor p53 in dormant tumours, while p53 inactivation alleviated dormant cell behaviours and enabled the exit of tumour mass dormancy. Additionally, dormant tumours were found to evolve within a permissive microenvironment associated with aberrant fibrosis and infiltration of activated matrix cancer-associated fibroblasts. Taken together, the results indicate that our β1 integrin-deficient GEMM model recapitulates all stages of tumour mass dormancy, including induction, maintenance, and re-emergence, and that re-emergence from dormancy involves cell intrinsic and extrinsic alterations which facilitate both cancer cell survival and proliferation.

## Results

### Mammary epithelial specific deletion of β1 integrin impairs tumour initiation through a coordinated induction of cell cycle arrest, apoptosis and cellular senescence

**Fig. 1:**
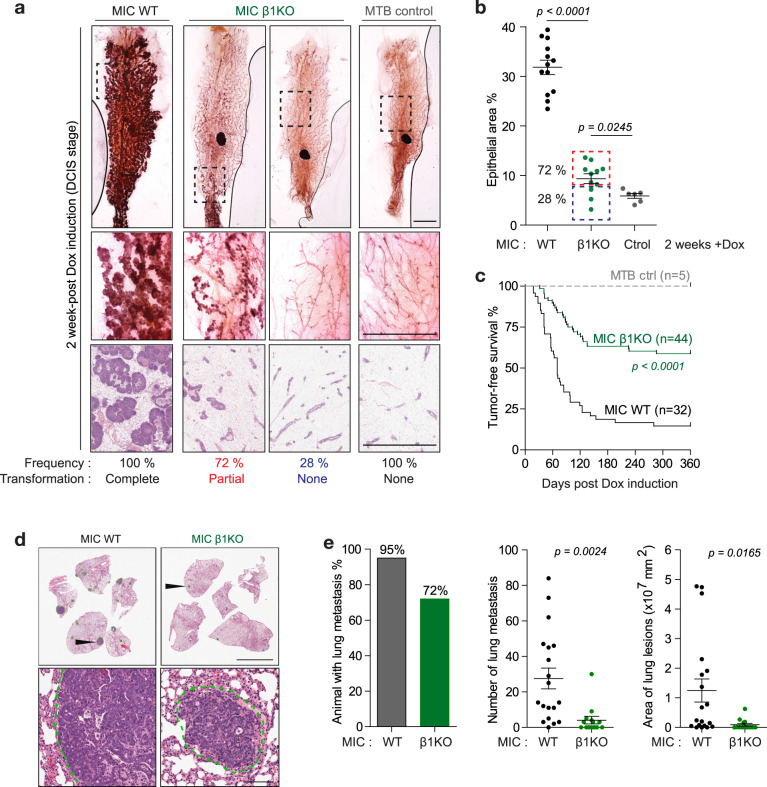
Mammary-specific ablation of β1 integrin inhibits tumour initiation, tumour progression to invasive carcinoma and lung metastasis. **a** Epithelial transformation (tumour initiation) after 2 week-post Dox induction in MIC mice carrying either wild type (MIC WT) or floxed β1 integrin (MIC β1KO) alleles. Upper panel shows whole mount analysis of mammary gland number 4. Scale bar is 5 mm. 28% of MIC β1KO mice did not exhibit epithelial transformation (similar to MTB control glands). Lower panel shows H&E images of hyperplastic lesions that are not yet invasive (MIN). Scale bars is 3 mm. MTB control mice represents non-transformed glands. **b** Quantification of epithelial area normalised to total gland area using H&E images in **a**. Data: mean ± SEM, two-tailed Student’s *t*-test. **c** Percent of tumour-free survival. *P* value was calculated between MIC WT and MIC β1KO cohort using Lox-rank Mantel Cox test. **d** Representative H&E images of lungs collected at end-burden tumour mass. Green outline indicates visible metastases. Scale bars are 1 cm (upper panel) and 100 μm (lower panel). **e** Quantification of lung metastatic burden including percentage of mice with metastasis (MIC WT *n* = 19, MIC β1KO *n* = 14), number and total area of lung metastases per animal. Data: mean ± SEM, two-tailed Student’s *t* test.

One major gap in our understanding of tumour dormancy is the lack of appropriate immune competent model that recapitulates the different stages of tumour dormancy. To address this issue, we generated the MIC GEMM that has several advantages over the conventional MMTV-PyV mT model (Fig. S1a) [11]. In the MIC model, PyV mT oncogene is coupled to Cre recombinase via an IRES element in the epithelial compartment (Fig. S1a) for their strict co-expression and deletion of conditional β1 integrin alleles in the presence of Doxycycline (Dox), and therefore eliminating ‘escapee’ tumours (Fig. S1c, d). Upon Dox induction, mammary tumours progress in a stepwise fashion ultimately leading to the metastatic colonisation of the lung (Fig. S1b). At 2 week-post Dox induction, 100% of MIC wild type (WT) mice developed ductal hyperplasia, a pathological stage referred to as MIN (mammary intra-epithelial neoplasm) (Fig. 1a, b). By contrast, deletion of β1 integrin significantly impairs mammary epithelial transformation, resulting in complete blockade in 28% of animals and partial transformation (limited transformation in number of mammary ducts) in the remaining animals (Fig. 1a, b). Impaired tumour initiation was further correlated with delayed tumour onset and reduced disease penetrance, from 85% in wild type cohort to 42% in β1 integrin-deficient cohort (Fig. 1c). These data indicate that out of 72% of β1 integrin-deficient animals that developed pre-neoplastic lesions, only half of them developed overt palpable tumours while the remaining half completely regressed. Furthermore, the tumours that developed in the absence of β1 integrin exhibited substantially reduced lung metastasis, including penetrance, number, and area of metastases (Fig. 1d, e). Taken together, these results show that β1 integrin loss significantly impairs mammary tumourigenesis and that its activity is required for transition from normal to invasive carcinoma.

**Fig. 2:**
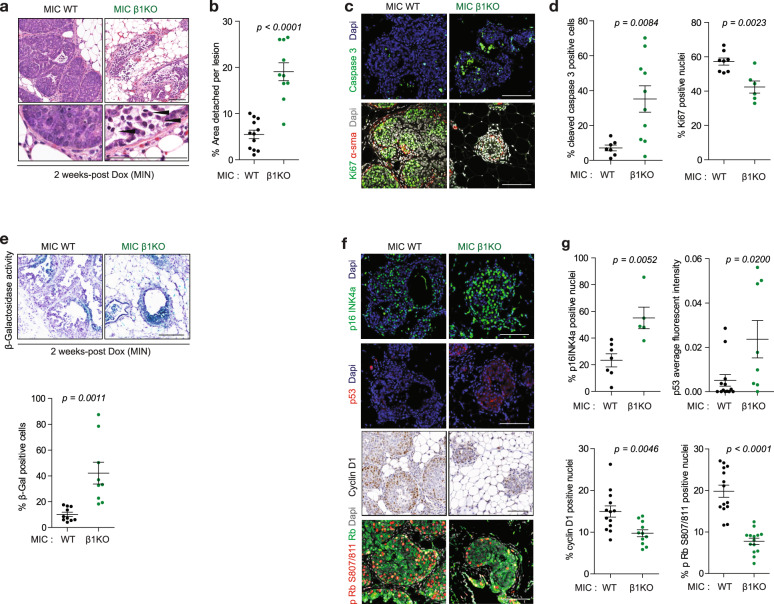
β1 integrin deletion results in cell detachment, proliferative block, apoptosis and cellular senescence in vivo. **a** H&E images of 2 week-post Dox induction MIN structures illustrating epithelial cell detachment from basement membrane. Arrow heads indicate dense apoptotic bodies or cells detaching into lumen area. **b** Quantification of area of detached epithelial from basement membrane normalised to the total area of basement per MIN structure. **c** Immunohistochemical staining of apoptotic marker (cleaved caspase 3, ccp3) and proliferative marker (Ki67). α-sma positivity indicates myoepithelial layer in MIN structure. Scale bar is 100 μm. **d** Percentages of ccp3+ and Ki67+ cells from images in **c**. **e** Representative images of MIN lesions stained for senescence-associated β-Galactosidase activity (SA-β-Gal, blue signal). Tissue is counterstained with Hematoxylin (purple signal). Scale bar is 100 μm. **f** Immunohistochemical staining of p16Ink4a, p53, cyclin D1 and phosphorylated Rb (S807/811) in MIN lesions. Scale bars are 100 μm. **g** Percentages of positive nuclei were quantified from images in **f**. Data: mean ± SEM, two-tailed Student’s *t* test.

To further evaluate the effects of β1 integrin ablation on early stages of mammary tumourigenesis, histopathological analyses of MIN structures at 2 week-post Dox induction revealed solid adenoma lesions in wild type MIC strain, yet epithelial cell detachment from the basement membrane and shedding of epithelial cells in the lumen of β1-deficient glands (Fig. 2a, b). This phenotype is consistent with the role β1 integrin receptors play in facilitating adhesion to the collagen-rich basement membrane [5]. In agreement with this hypothesis, we confirmed that β1 integrin-deficient MIC epithelial cells were unable to attach to Collagen I in vitro (Fig. S1e). To further assess the proliferative and apoptotic capacities, we performed immunohistochemical (IHC) staining with both Ki67 and cleaved caspase 3, respectively, and observed that the reduction of epithelial transformation in β1 integrin-deficient glands was associated with a decrease in epithelial proliferation and increase in apoptotic cell death (Fig. 2c, d). In addition to its impact on cell proliferation and apoptosis, integrin signalling has been implicated in supressing cellular senescence [12], prompting us to measure the levels of senescence-associated β-Galactosidase activity. The results showed that loss of β1 integrin resulted in dramatic elevation of senescence-associated β-Galactosidase activity (Fig. 2e). The control of cell proliferation, cell senescence and apoptotic programmes is ultimately governed by a number of key cell cycle regulators and tumour suppressor check points [13, 14]. Indeed, IHC analyses confirmed that the proliferation defect seen in β1 integrin-deficient MIC mammary glands was correlated with reduction in both Cyclin D1 and phospho-Rb and an increase in p16Ink4 levels (Fig. 2f, g). Notably, an increase in levels of p53 tumour suppressor was further correlated with both senescence and apoptotic phenotypes observed in the β1-deficient mammary glands (Fig. 2f, g). Together these results argue that massive decrease in hyperplastic lesions is associated with coordinated increase in epithelial cell apoptosis, senescence, and cell cycle arrest due to activation of these key tumour suppressor-mediated check-points.

**Fig. 3:**
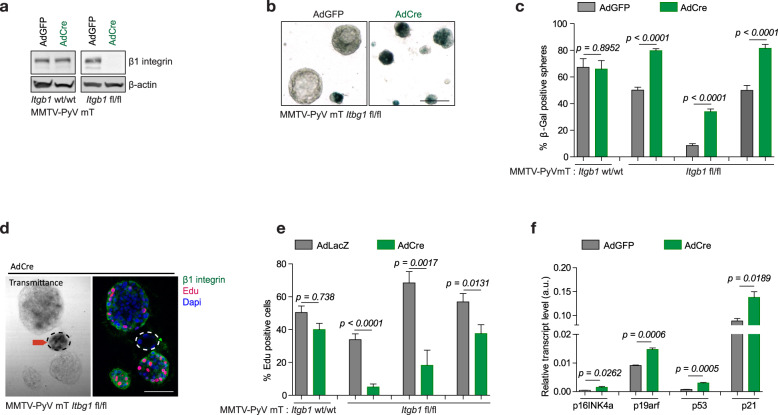
β1 integrin deletion promotes in cell cycle impairment and cellular senescence in established cell lines. **a** Immunoblot analysis of MMTV-PyV mT cells carrying either wild type β1 integrin alleles (Itbg1^wt/wt^) or conditional floxed alleles (Itbg1^fl/fl^) at day 4 post-AdGFP or AdCre viral infection with loading control (β-actin). **b** Senescence-associated β-Galactosidase activity in cells at day 8 post-AdGFP or AdCre viral infection. Scale bar is 100 μm. **c** Percentages of spheres positive for senescence-associated β-Galactosidase activity for the indicated cell lines (one Itbg1^wt/wt^ cell line and three Itbg1^fl/fl^ cell lines). **d** Tumour spheres (day 8-post AdLacZ or AdCre) were stained for Edu incorporation, β1 integrin expression and senescence-associated β-Galactosidase activity. X-Gal fluorescence was captured by confocal microscopy, excitation at 633 nm. Arrow indicates a tumour sphere devoid of β1 integrin (successful AdCre infection and β1 integrin knockout) and lacking Edu incorporation. At least ten spheres were quantified. Scale bar is 100 μm. **e** Percentages of Edu+ nuclei per sphere. Only spheres lacking β1 integrin expression were quantified for AdCre condition. Data are shown for one Itbg1^wt/wt^ cell line and three independent Itbg1^fl/fl^ cell lines. **f** Transcript levels were averaged from quadruplicate for indicated genes by RT-qPCR. The error bars indicate ±SD, two-tailed Student’s *t* test.

Although these results provide compelling evidence for the requirement of β1 integrin in the initiation phase of mammary tumourigenesis, whether β1 integrin is required for maintenance of the transformed state was unclear. To address this issue, we employed an Adenoviral Cre vector system to ablate β1 integrin expression in vitro. Using several primary cancer cell lines derived from MMTV-PyV mT tumours bearing either wild type (wt/wt) or homozygous conditional (fl/fl) alleles for β1 integrin, we achieved efficient β1 integrin ablation in the latter (Fig. 3a). We subsequently demonstrated that loss of β1 integrin in tumour spheroids grown in 3D matrix was associated with cellular senescence (Fig. 3b, c), diminished Edu incorporation for cell division (Fig. 3d, e) and a substantial reduction in size (Fig. S1f). Parallel to the in vivo studies, loss of β1 integrin function in tumour spheroids was further correlated with upregulation of p16Ink4a, p16arf, p53 and p21 transcripts (Fig. 3f). Collectively, these data indicate that β1 integrin receptors are required for both tumour initiation and maintenance through suppressing p53-dependent tumour suppressor pathways.

### Mammary epithelial loss of p53 tumour suppressor bypasses tumour dormancy imposed by β1 integrin deficiency

**Fig. 4:**
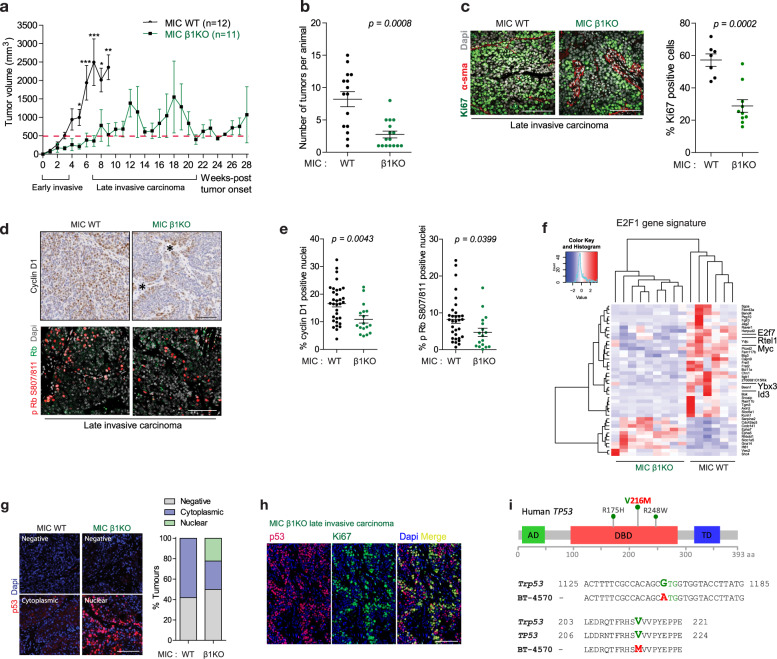
β1 integrin-deficient tumours exhibit tumour mass dormancy associated with p53 gene alteration. **a** Tumour volume was measured from tumour onset to end-burden. *P* values (* <0.05, ** <0.01, *** <0.001) were calculated using 2-way ANOVA test. Red line indicates threshold between early (<500 mm^3^) and late invasive carcinomas (>500 mm^3^) collected at end-burden. **b** Number of tumours at end-burden. Data ± SEM, two-tailed Student’s *t*-test. **c** Analysis of Ki67 of cancer cells in late invasive carcinoma. α-sma indicates stromal area. Scale bar is 100 μm. Data ± SEM, two-tailed Student’s *t* test. **d** Analysis of cyclin D1 and phosphor-Rb (S807/811) in late invasive tumours. Asterisks indicate stromal area. Tumour cells with high cyclin D1 expression localise adjacent to stroma regions in MIC β1KO late invasive carcinomas. Data ± SEM, two-tailed Student’s *t* test. Scale bar is 100 μm. **e** Quantification of cyclin D1 and phosphor-Rb from images in **d**. Data ± SEM, two-tailed Student’s *t* test. **f** DEGs in end-burden tumours are overlapped with a E2F1 ChIP-seq dataset to generate a E2F1 gene signature. Several E2F1 target genes (*E2f7*, *Rtel1*, *Myc*, *Ybx3*, *Id3*) that are directly involved in cell cycle regulation are downregulated in MIC β1KO tumours. **g** Immunohistochemical analysis of p53 in late invasive carcinoma (MIC WT *n* = 22, MIC β1KO *n* = 14). Left, representative of different p53 staining patterns—absent, weak cytoplasmic or strong nuclear. Right, percentage of tumour with distinct p53 distribution pattern. **h** Co-staining of MIC β1KO tumours (*n* = 2) with nuclear p53 and proliferation marker Ki67. Cells with nuclear p53 are also Ki67 positive. **i** Sanger sequencing of p53 in a reactivated MIC β1KO tumour (ID BT-4570) identifies a G-A substitution in *Trp53* exon 6. The mutation is mapped to V216M (Valine to Methionine) located in DNA-binding domain of p53 and conserved between mouse and human.

Next, we assessed the importance of β1 integrin during the pathological transition from non-invasive ductal hyperplasia (MIN) to invasive carcinoma in later stages of the disease (Fig. S2a, b). Overall, our data indicated that progression of tumours to late invasive stage is severely compromised by loss of β1 integrin function as evidenced by a decreased penetrance (43% vs 85%) of palpable tumours (Fig. 1c), decrease in tumour volume (Fig. 4a) and number of palpable tumours (Fig. 4b). Consistent with smaller tumour volumes exhibited by β1 integrin-deficient mice (Fig. 4a), assessment of growth rate of palpable individual β1 integrin-deficient lesions revealed two distinct behaviours: one group with maintained a stable tumour mass for the experimental time frame while others with the ability to reactivate tumour growth (Fig. S2c).

To further evaluate the basis for the variable tumour behaviour exhibited by β1 integrin-deficient lesions, IHC analyses showed that emerging tumours had dramatic reduced levels of key proliferation markers Ki67, cyclin D1 and phospho-Rb (Fig. 4c–e), which is consistent with the early β1 integrin-deficient lesions (Fig. 2c, d). Whole tumour RNA-sequencing revealed drastic alteration of tumour transcriptome (Fig. S3a), with 238 differentially expressed genes (DEGs): 107 upregulated and 121 downregulated transcripts in β1 integrin-deficient tumours compared to the wild type counterparts (Fig. S3a). Ingenuity pathway analysis (IPA) of the DEGs revealed several candidate regulators that are either inhibited or activated upon β1 integrin loss, including a E2F1 inactivation signature (Fig. S3b). Cross comparison of our DEGs to a E2F1 cistrome revealed many E2F1 target genes (*E2f7*, *Rtel1*, *Myc*, *Ybx3* and *Id3*) being downregulated in β1 integrin-deficient tumours, consistent with slow tumour growth phenotype (Fig. 4f).

In addition to E2F1 inhibition, IPA analyses of RNA-seq also predicted a signature of p53 activation associated with the transcriptome of β1 integrin-deficient tumours (Fig. S3b). Given this observation, IHC analyses of p53 on recurrent β1 integrin-deficient tumours indicated a distinct staining pattern between β1 integrin-proficient and -deficient tumours, where 22% of the β1 integrin-deficient tumours (3 out of 14 tumours) exhibited high nuclear p53 positivity (Fig. 4g). Given that strong p53 immunostaining of tumour tissue was reported to be predictive of *TP53* mutations in various human cancers, including breast carcinoma [15] we evaluated whether p53 positive tumour cells were actively proliferating by performing IHC analysis of a proliferative marker Ki67. The results revealed that p53 positive cell were actively proliferating, arguing they possessed loss of function p53 mutations (Fig. 4h). Using additional β1 integrin-deficient tumours that were not profiled by RNA-seq, we performed direct p53 gene sequencing by Sanger method and identified a missense mutation (14%, 1 out of 7 tumours) resulting in Val/Met substitution in exon 6 (Fig. 4i). This mutation is mapped to an analogous V216M mutation in human, located alongside other well-characterised p53 mutations (R175H, R248W) in the hotspot DNA binding domain (Fig. 4i). Similar to those mutations, V216M mutation has also been found in several cancer types and associated with loss of function as evidenced by 90% reduction in transactivation capacity [16, 17]. Collectively these data argue that exit from tumour dormancy in a portion of the tumours correlates with inactivation of p53 tumour suppressor pathway.

**Fig. 5:**
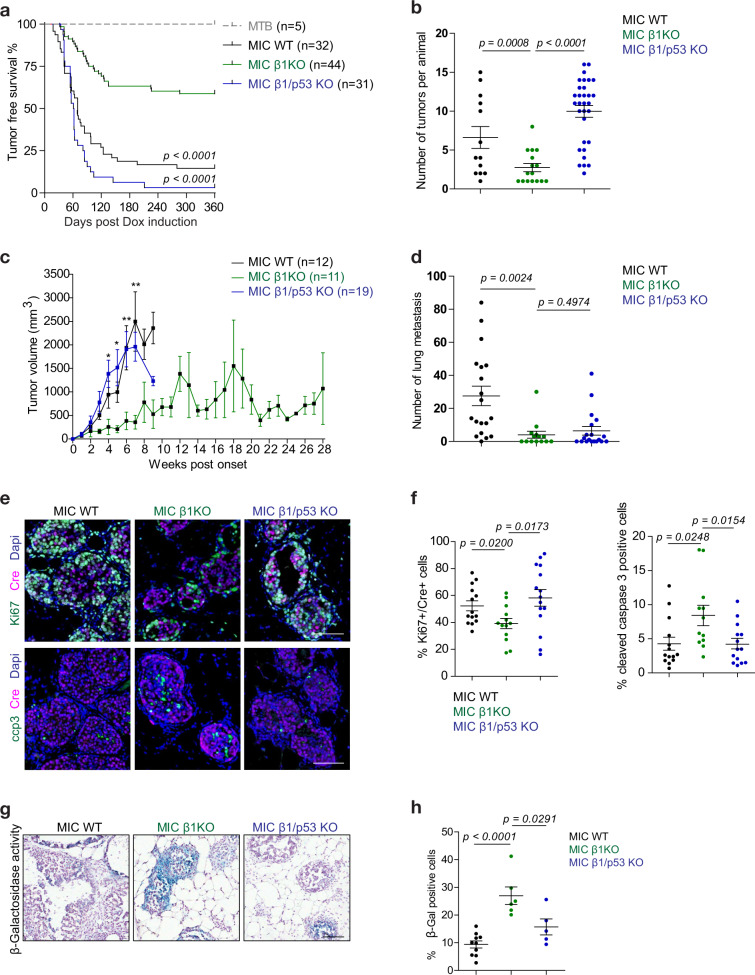
Genetic inactivation p53 rescues tumourigenesis and cancer dormancy driven by β1 integrin deficiency. **a** Percent of tumour-free survival. *P* values (Lox-rank Mantel Cox) were calculated between MIC β1KO cohort to MIC WT, or to MIC β1/p53 KO cohort. Data were extended from Fig. 1c. **b** Number of individual tumours at end-burden. **c** Tumour volume was measured for the indicated genotypes. *P* values were calculated between MIC β1KO and MIC β1/p53 KO pair using 2-way ANOVA test. *P* values * <0.05, ** <0.01. **d** Number of lung metastatic lesions per animal. **e** Analysis of Ki67 and cleaved caspase 3 (ccp3) in pre-invasive MIN lesions at 2 week-post Dox induction. Cre expression indicates transgenic cells. Scale bar is 100 μm. **f** Quantification of Ki67+ and ccp3+ nuclei in transgenic Cre+ cell population from images in **e**. **g** Senescence-associated β-Galactosidase activity (blue signal) assay in 2 week-post Dox induction MIN structures. Tissue is counterstained with Hematoxylin (purple signal). Scale bar is 100 μm. **h** Average of senescence-associated β-Galactosidase activity-positive cells per animal. For all data, the centre line indicates the mean and error bars indicate ±SEM, two-tailed Student’s *t* test.

To functionally validate the role of p53 pathway in regulating cancer dormancy, we introduced conditional p53 alleles into β1 integrin-deficient mice [18]. Pathological analysis of early tumour progression (2 week-post Dox induction) indicated that inactivation of p53 was unable to restore the amount of epithelial transformation that loss of β1 integrin imposes, arguing that the initial effects of β1 integrin function on early tumourigenesis are independent of p53 function (Fig. S4a, b). However, progression to invasive carcinoma appears to be absolutely dependent on p53 as its inactivation in β1 integrin-deficient mice completely restored tumour onset, penetrance, and tumour multiplicity to comparable levels as MIC WT counterparts (Fig. 5a, b). With regards to tumour mass dormancy, we monitored p53/β1 integrin-deficient mice and found that loss of p53 fully restored the defective growth rate due to β1 integrin deletion (Fig. 5c), yet p53 inactivation was unable to restore the reduced metastatic potential of β1 integrin-deficient cancer cells (Figs. 5d, S4c–e). Overall, these data support a functional link between β1 integrin and p53, in which β1 integrin-dependent suppression of p53 is required for breast cancer progression. Furthermore, the inability of p53 loss to rescue initial hyperplastic transformation or metastatic dissemination clearly indicates that β1 integrin controls additional cellular activities that are independent of p53 function (Figs. S4a-e, 5d).

To further understand how p53 pathway regulate the transition from non-invasive to invasive carcinoma in the absence of β1 integrin, analysis of pre-invasive hyperplastic lesions at 2 week-post Dox induction for cell proliferation, apoptosis and cellular senescence showed that deletion of p53 restored the proliferative defect and abrogated the increased cell death exhibited by β1 integrin deficient lesions (Fig. 5e, f). MIN structures lacking p53 expression also display restoration of cyclin D1 level and Rb activity (S807/811 phosphorylation) which in turn correlates with increase in cell proliferation (Fig. S4f, g). Consistent with importance of p53 in cell senescence, p53 inactivation resulted in a profound block in cellular senescence (Fig. 5g, h). Taken together, these data demonstrate that bypassing p53-dependent senescence is a key event in exiting tumour mass dormancy in absence of β1 integrin function. (Fig. 5c, g, h).

### Evolution of β1 integrin-deficient dormant tumours is associated with the remodelling of tumour microenvironment

**Fig. 6:**
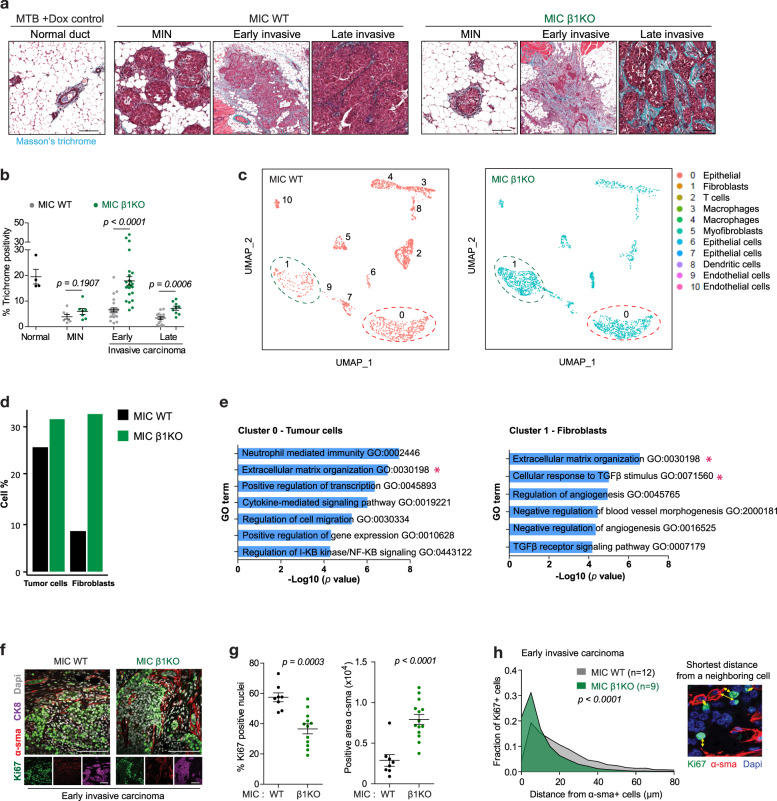
β1 integrin-deficient dormant tumours evolve within an active tumour microenvironment enriched with abundant ECM and CAFs. **a** Masson trichrome staining to visualise collagen deposition (green signal) in normal duct, MIN, early and late invasive carcinoma. All scale bars are 100 μm. **b** Quantification of collagen positive area for the indicated stages and genotypes in **a**. **c** Single cell RNA sequencing of early invasive carcinoma from MIC WT lesions (fast growing) or MIC β1KO lesions (dormant). UMAP analysis reveals 11 clusters, including cancer and stromal populations (3281 cells/MIC WT lesions and 1768 cells/MIC β1KO lesions). **d** Percentage of cancer cell clusters (cluster 0, expressing PyV mT and/or Cre recombinase) and fibroblast clusters (cluster 1) from UMAP analysis. **e** Gene Ontology (GO) analysis of differentially expressed genes between MIC WT and MIC β1KO for cancer cell and fibroblast clusters. Asterisks indicate gene signatures enriched for ECM processes. **f** Immunohistochemical analyses of early invasive carcinoma for Ki67, α-sma (CAF marker) and CK8 (mammary tumour cell marker). Scale bar is 100 μm. **g** Quantification of percentage of Ki67+ in CK8 + cell population and amount of α-sma+ fibroblast cell infiltration to early carcinoma lesions. **h** Analysis of distance from a proliferative cancer cell (Ki67+/CK8+) to the nearest fibroblast (α-sma+) from images in **f**. Right, example of nearest distances. Left, quantification of total 5505 cells from MIC WT (total 12 lesions) and 7651 cells from MIC β1KO (total 9 lesions). *P* value was calculated using averaged distance for each lesion, Student’s *t* test. For all data, the centre line indicates the mean and error bars indicate ± SEM, two-tailed Student’s *t* test.

Although our observations argue that tumour cell autonomous mutations in p53 suppressor pathway are involved in exit from tumour dormancy, another important event that coincides with the evolution of β1 integrin-deficient tumours is the extensive remodelling of the tumour microenvironment (TME). Of particular note, β1 integrin-deficient tumours exhibited a dramatic increase in stromal content that correlated with massive deposition of collagen at later stages of tumour evolution (Fig. 6a, b) and epithelial cells positive for proliferation markers were spatially localised next to its adjacent stroma (Fig. 4c–e), altogether indicating a paracrine relationship between cancer cells and its activated TME. Consistent with these pathological observations, Gene Ontology (GO) analysis of these tumours also confirmed upregulation of genes involved in ECM synthesis, ECM organisation and connective tissue development (Fig. S5b).

To gain further molecular insight underlying this TME remodelling, we performed single cell RNA sequencing (scRNA-seq) on pooled samples derived from early invasive carcinoma from β1 integrin-proficient (3281 cells, with a median of 8376 unique molecular identifiers (UMIs) and 2609 genes per cell) and β1 integrin-deficient dormant lesions (2768 cells, with a median of 12,378 UMIs and 3530 genes per cell) (Fig. S6a, b). Uniform Manifold Approximation and Projection were used to assign cells into distinct clusters based on their transcriptomic similarities and both samples were partitioned into 11 clusters (Fig. 6c). Cluster 0 was identified as transgenic cancer cells with expression of PyV mT oncogene and Cre recombinase (Fig. S6c) and the identities of stromal clusters were assigned based on their transcriptional profiles (Fig. S7). Bioinformatics analyses revealed major changes in stromal cell composition of β1 integrin-deficient lesions, including decrease in immune cells (cluster 2, 3, 4) and increase in endothelial cells (cluster 9) (Figs. 6c and S6d). Notably, β1 integrin-deficient lesions exhibit a 4-fold increase in matrix fibroblast cells (cluster 1) that are characterised by transcriptome associated with mesenchymal cells and highest expression of ECM-related genes (*Col*, *Mmp*, *LoxL, Cxcl12*) (Figs. 6d and S7). Using GO term analysis to compare the transcriptomes between β1 integrin-proficient and -deficient lesions, we observed that cancer-associated fibroblasts (CAFs) in dormant lesions have differentially activated ECM processes and TGF-β stimulus (Fig. 6e), corroborating with previous reports indicating mesenchymal stromal cells as a precursor of CAFs and that their transition into CAFs is dependent on TGF-β signalling [19, 20]. In addition, GO term analysis of tumour cell (cluster 0) transcriptome in β1 integrin-deficient lesions also indicated upregulation genes involved in ECM-related processes, suggesting that both cancer cells and CAFs are responsible for the TME remodelling observed throughout dormancy period (Fig. 6a, e).

To further confirm our bioinformatic observations, IHC staining of smooth muscle actin (α-sma) on early invasive β1-deficient lesions showed a substantial increase in CAF infiltration (Fig. 6f, g), agreeing with increased ECM deposition. As α-sma is expressed not only in CAFs but also in myoepithelial cells, we performed co-staining of α-sma and a myoepithelial marker CK14 and confirmed the increase in CAF population (α-sma+/CK14-) (Fig. S5f, g). Using another CAF marker, Desmin, we further show that β1 integrin-deficient tumours also display a marked increase in stromal Desmin+ cells (Fig. S5h, i) [21]. Furthermore, IHC analysis of early invasive lesions for Ki67 revealed distinct distribution of proliferative cells in relation to α-sma+ CAFs. A small fraction of β1 integrin-deficient cancer cells that are proliferative reside strictly adjacent to α-sma+ CAFs (Fig. 6f). Quantification of the distance from Ki67+/CK8 + cancer cells to the nearest α-sma+ CAF confirms the reduced proximity in lesions lacking β1 integrin expression in comparison to wild type lesions (Fig. 6h). We also observed this extensive ECM modelling in β1 integrin-deficient lesions that resumed tumour growth and progressed into late invasive carcinoma, arguing the importance of CAFs during the exit phase of dormancy (Fig. S5c, d).Given that the pro-tumourigenic role of CAFs is well established through their ability to create a permissive TME by ECM remodelling and cytokine secretion [20], we hypothesised that the accumulation CAFs during tumour dormancy period promotes dormant β1 integrin-deficient cells to regain proliferative capacity.

## Discussion

It is well established that integrin crosstalk with growth factor receptors plays an important role in transducing environmental cues and in influencing numerous aspects of malignant progression [6, 22]. We previously demonstrated that mammary epithelial ablation of β1 integrin resulted in profound delay in tumour induction associated with a phenotype characteristic of tumour dormancy [7]. However, because the tumours that arose in this model retained β1 integrin function due to their escape from Cre-mediated recombination, understanding the mechanism of dormancy exit was limited. In this study, using a coupled inducible PyV mT-IRES-Cre GEMM, we demonstrated that while mammary epithelial specific disruption of β1 integrin severely impairs mammary tumour progression through induction of tumour mass dormancy (Fig. 1), β1 integrin-deficient tumours can eventually arise. Biochemical and genetic analyses further demonstrated that these β1 integrin-deficient tumours likely evolve through inactivation of p53 tumour suppressor and through remodelling of the tumour microenvironment.

Breast cancer progression is a multistep process involving distinct pathological stages and adaptation to support tumour growth and metastatic dissemination. Although acquisition of genetic alterations underlies initiating events and loss of cellular homoeostasis, cells with perturbed homoeostasis due to such oncogenic events are often kept in check by growth arrest, cell death and host immunity. Overcoming these tumour suppressive barriers is a crucial step in permitting transition from benign lesions to malignant carcinomas. Here, we show that β1 integrin receptor signalling plays a pivotal role in restraining these initial barriers to enable tumour growth. Epithelial-specific deletion of β1 integrin results in detrimental defects in serval cellular processes including loss of cell adhesion (Fig. 2a) and cell cycle exit (Fig. 2c) that are correlated with increased in cell death (Fig. 2c) and cellular senescence (Fig. 2e). Similar observations were made using established MMTV-PyV mT cells derived from end-burden tumours and AdCre system (Fig. 3). Although the induction of apoptosis was noted upon β1 integrin deletion in transition from normal epithelial to MIN stage (Fig. 2c), deletion of β1 integrin in established PyV mT tumour cells resulted in cell cycle arrest associated with cellular senescence (Fig. 3) without overt evidence of apoptotic cell death [7]. Collectively, these data argue that apoptotic checkpoints in established cancer cells are no longer dependent on β1 integrin function.

Molecular and bioinformatics analyses also indicate that β1 integrin loss can elicit activation of p53, a key tumour suppressor that can directly attribute to cell cycle impairment, senescence and cell death (Figs. 2 and 4f). Indeed, several re-activated β1-integrin deficient tumours acquired genetic alterations that disrupt *Trp53* gene functions (Fig. 4g-i). We functionally validated its importance in β1 integrin loss-induced dormancy exit by showing that epithelial disruption of p53 rescued tumour formation in these β1 integrin-deficient mice (Fig. 5). And that these tumours lack the extensive collagen deposition exhibited the β1 integrin-deficient tumours (Fig. S5a). The rescue of these dormant phenotypes was associated with decrease in tumour cell apoptosis, increase cell proliferative status (Fig. 5e, f) and suppression of cellular senescence at early stages of tumour progression (Fig. 5, h). Our observation liking integrin activity to p53-mediated cell death is also supported by a previous study demonstrating that p53 modulates survival signal from ECM/FAK in malignant cells [23]. Although p53 loss completely rescued tumour formation, abrogation of p53 did not result in re-acquisition of the metastatic phenotype (Fig. S4c–e) or the initial expansion of MIN lesions observed in parental MIC strain (Fig. S4a). These observations indicate the β1-integrin containing receptors have additional tumour suppressor-independent functions such as cell and cell substratum adhesion [5, 24] and formation of invadopodia that are key structures promoting invasive tumour phenotype [24].

In addition to cell intrinsic factors such as tumour suppressors that regulate dormancy, our β1 integrin-deficient mouse model revealed that emergence from tumour dormancy may also involve cancer cell non-autonomous tumour microenvironmental signals. Indeed, β1 integrin-deficient lesions that escaped dormancy possessed numerous stromal alterations reflecting fibrosis, starting with expression of ECM and fibroblast-recruiting genes in tumour epithelial cells (Figs. 6e and S5b), CAF infiltration (Fig. 6), and extensive ECM modelling. We hypothesise that the observed stromal activation is a consequence of senescence-associated secretory phenotype, including a vast array of secreted factors that can influence the surrounding stromal cell types such as fibroblasts, endothelial and immune cells [25]. This process also requires the timeframe provided by dormancy in β1 integrin-deficient tumours to allow for stromal remodelling, which fast-growing tumours do not display (Fig. S5a), and for selection of p53 genetic inactivation, collectively permitting dormancy exit. Conceivably, these data reflect the dependence of epithelial cell proliferation on the adjacent stromal cells at early-stage tumour invasion and that fibrosis is an integral component of tumour mass dormancy. Congruently, we showed that the proliferative β1 integrin-deficient epithelial cell population were in close proximity to CAFs (Figs. 6, h, S5c, d). Furthermore, sc-RNA-seq analyses of dormant lesions also revealed alteration in other stromal populations, notably a reduction in immune cell clusters (cluster 2, 3, and 4) (Fig. S6d), suggesting involvement of immune surveillance during tumour mass dormancy. Indeed, PyV mT-driven mammary tumourigenesis in MIC model has been shown to engage a highly active immune TME modulated by Stat3 transcription factor [26]. We propose that the decrease in T cell and macrophage infiltration may allow dormant β1 integrin-deficient tumour cells to remain undetectable, serving as a prerequisite for dormant tumours to exit dormancy. Collectively these data argue at in the early stage of dormancy, β1 integrin-deficient epithelial cells require a permissive TME before intrinsic mutations to allow dormancy exit.

**Fig. 7:**
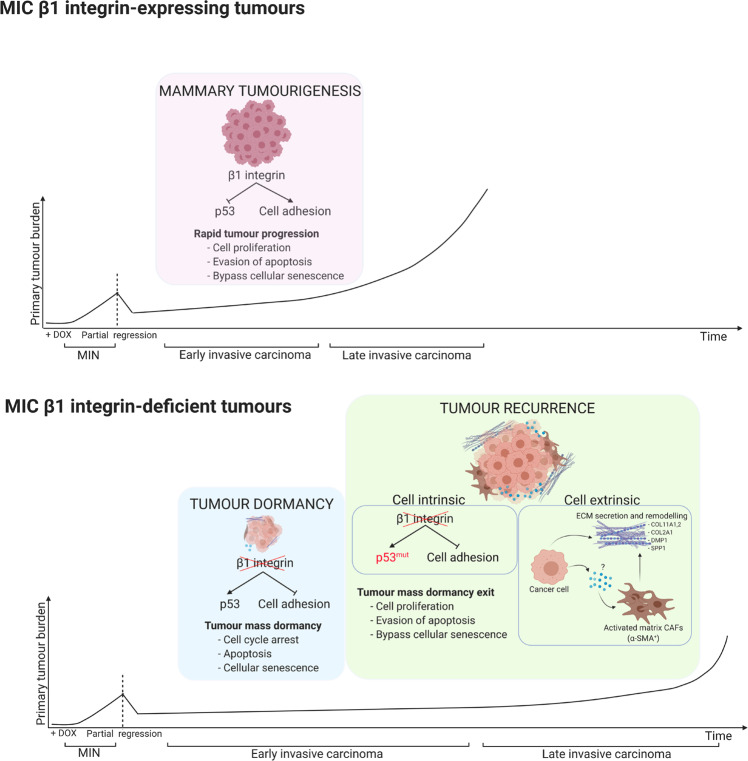
Schematic model of tumour mass dormancy induction and exit phase regulated by β1 integrin. Schematic model highlighting the roles of β1 integrin receptors in regulating tumour mass dormancy induction and exit phase. In MIC WT model, β1 integrin suppresses p53 and Rb activities thereby allowing evasion of tumour suppressive barriers including cell cycle arrest, apoptosis, and cellular senescence. In contrast, β1 integrin deficiency reactivates those suppressive barriers, resulting the induction tumour mass dormancy. Tumour recurrence from dormancy exit requires both cell intrinsic (p53 alteration) and extrinsic events (CAF infiltration and ECM deposition). Potential factors mediating paracrine communication between cancer cells and fibroblasts are remained to be addressed. Created with BioRender.com.

The dependence of ECM remodelling in β1 integrin-deficient tumours reflects the key role of these heterodimeric collagen receptors in pathological studies of human breast cancer tissues and their malignant progression [27]. Particularly, increases in collagen deposition, crosslinking and stiffening occur as early as at the MIN stage and continue during malignant progression to metastatic stage [27, 28]. High mammographic density is also associated with increased collagen deposition and is one of the strongest risk factors for the development of invasive carcinoma [29]. The functional importance for collagen-modifying enzyme LOX [6] and MMP9 [30] and integrin-associated signalling molecules such as FAK [9], c-Src [8] and ILK [10] have been implicated in driving mammary tumourigenesis in a number of studies. Additionally, similar observations regarding the importance of ECM/integrin signalling have also been made in the context of other oncogenes, such as human HER2 receptor, reiterating that ECM/β1 integrin axis plays a key cooperative function with aberrant growth signals to drive breast cancer malignancy [31, 32]. Taken together, these observations argue that activation of β1 containing integrin receptors and their coupled downstream signalling pathways play a critical role in tumourigenesis through suppressing tumour cell autonomous checkpoints such as p53 as well as modulating the adjacent ECM microenvironment (Fig. 7). The future identification of integrin-dependent paracrine signals involved in modulating the tumour microenvironment will provide critical insight in tumour dormancy.

## Materials and methods

### Animal models

Generation of MIC and MMTV-PyV mT transgenic mice was described previously ^8, 37^. Mice with floxed *Itgb1* (β1 integrin) alleles were obtained from Dr. Mueller and described elsewhere [33]. Mice with floxed *Tp53* alleles were obtained from the National Cancer Institute mouse depository and described elsewhere [34]. MMTV-reverse tetracycline transactivator (rtTA) transgenic mice were generated in the laboratory of Dr. Lewis Chodosh as previously described [35]. All mice were bred and maintained on the pure FVB background. Genomic DNA was extracted from tails of all experimental mice using crude salt extraction and subsequently used for genotype confirmation using PCR using the following primers: PyV mT - left primer: GGAAGCAAGTACTTCACAAGGG, right primer: GGAAAGTCACTAGGAGCAGGG; MTB - left primer: ACCGTACTCGTCAATTCCAAGGG, right primer: TGCCGCCATTATTACGACAAGC; Itgb1 - left primer: GCCGCCACAGCTTTCTGCTGTAGG, right primer: CTGATCAATCCAATCCAGGAAACC; Tp53 - left primer: CACAAAAACAGGTTAAACCCAG, right primer: AGCACATAGGAGGCAGAGAC. Experimental and control animals were given drinking water with Doxycycline (2 mg/mL) at 10 weeks of age and monitored weekly by physical palpations for tumour formation. All mice were housed and handled at the Comparative Medicine and Animal Resource Tumour in accordance with McGill University Animal Ethics Committee guidelines.

## Supplementary information

Supplementary information

Supplementary table 1

## References

[1] Phan TG, Croucher PI (2020). The dormant cancer cell life cycle. Nat Rev Cancer.

[2] Triana-Martinez F, Loza MI, Dominguez E (2020). Beyond tumor suppression: senescence in cancer stemness and tumor dormancy. Cells.

[3] Barkan D, Green JE, Chambers AF (2010). Extracellular matrix: a gatekeeper in the transition from dormancy to metastatic growth. Eur J Cancer.

[4] Pontier SM, Muller WJ (2008). Integrins in breast cancer dormancy. APMIS.

[5] Desgrosellier JS, Cheresh DA (2010). Integrins in cancer: biological implications and therapeutic opportunities. Nat Rev Cancer.

[6] Levental KR, Yu H, Kass L, Lakins JN, Egeblad M, Erler JT (2009). Matrix crosslinking forces tumor progression by enhancing integrin signaling. Cell.

[7] White DE, Kurpios NA, Zuo D, Hassell JA, Blaess S, Mueller U (2004). Targeted disruption of beta1-integrin in a transgenic mouse model of human breast cancer reveals an essential role in mammary tumor induction. Cancer Cell.

[8] Marcotte R, Smith HW, Sanguin-Gendreau V, McDonough RV, Muller WJ (2012). Mammary epithelial-specific disruption of c-Src impairs cell cycle progression and tumorigenesis. Proc Natl Acad Sci USA.

[9] Lahlou H, Sanguin-Gendreau V, Frame MC, Muller WJ (2012). Focal adhesion kinase contributes to proliferative potential of ErbB2 mammary tumour cells but is dispensable for ErbB2 mammary tumour induction in vivo. Breast Cancer Res.

[10] Pontier SM, Huck L, White DE, Rayment J, Sanguin-Gendreau V, Hennessy B (2010). Integrin-linked kinase has a critical role in ErbB2 mammary tumor progression: implications for human breast cancer. Oncogene..

[11] Rao T, Ranger JJ, Smith HW, Lam SH, Chodosh L, Muller WJ (2014). Inducible and coupled expression of the polyomavirus middle T antigen and Cre recombinase in transgenic mice: an in vivo model for synthetic viability in mammary tumour progression. Breast Cancer Res.

[12] Kren A, Baeriswyl V, Lehembre F, Wunderlin C, Strittmatter K, Antoniadis H (2007). Increased tumor cell dissemination and cellular senescence in the absence of beta1-integrin function. EMBO J.

[13] Moreno-Layseca P, Streuli CH (2014). Signalling pathways linking integrins with cell cycle progression. Matrix Biol.

[14] Jones MC, Zha J, Humphries MJ (2019). Connections between the cell cycle, cell adhesion and the cytoskeleton. Philos Trans R Soc Lond B Biol Sci.

[15] Murnyak B, Hortobagyi T (2016). Immunohistochemical correlates of TP53 somatic mutations in cancer. Oncotarget..

[16] Grochova D, Vankova J, Damborsky J, Ravcukova B, Smarda J, Vojtesek B (2008). Analysis of transactivation capability and conformation of p53 temperature-dependent mutants and their reactivation by amifostine in yeast. Oncogene..

[17] Zerdoumi Y, Lanos R, Raad S, Flaman JM, Bougeard G, Frebourg T (2017). Germline TP53 mutations result into a constitutive defect of p53 DNA binding and transcriptional response to DNA damage. Hum Mol Genet.

[18] Marino S, Vooijs M, van Der Gulden H, Jonkers J, Berns A (2000). Induction of medulloblastomas in p53-null mutant mice by somatic inactivation of Rb in the external granular layer cells of the cerebellum. Genes Dev.

[19] Kojima Y, Acar A, Eaton EN, Mellody KT, Scheel C, Ben-Porath I (2010). Autocrine TGF-beta and stromal cell-derived factor-1 (SDF-1) signaling drives the evolution of tumor-promoting mammary stromal myofibroblasts. Proc Natl Acad Sci USA.

[20] Sahai E, Astsaturov I, Cukierman E, DeNardo DG, Egeblad M, Evans RM (2020). A framework for advancing our understanding of cancer-associated fibroblasts. Nat Rev Cancer.

[21] Bartoschek M, Oskolkov N, Bocci M, Lovrot J, Larsson C, Sommarin M (2018). Spatially and functionally distinct subclasses of breast cancer-associated fibroblasts revealed by single cell RNA sequencing. Nat Commun.

[22] Hamidi H, Ivaska J (2019). Author Correction: Every step of the way: integrins in cancer progression and metastasis. Nat Rev Cancer.

[23] Ilic D, Almeida EA, Schlaepfer DD, Dazin P, Aizawa S, Damsky CH (1998). Extracellular matrix survival signals transduced by focal adhesion kinase suppress p53-mediated apoptosis. J Cell Biol.

[24] Peláez R, Pariente A, Pérez-Sala Á, Larrayoz IM (2019). Integrins: moonlighting proteins in invadosome formation. Cancers.

[25] Faget DV, Ren Q, Stewart SA (2019). Unmasking senescence: context-dependent effects of SASP in cancer. Nat Rev Cancer.

[26] Jones LM, Broz ML, Ranger JJ, Ozcelik J, Ahn R, Zuo D (2016). STAT3 establishes an immunosuppressive microenvironment during the early stages of breast carcinogenesis to promote tumor growth and metastasis. Cancer Res.

[27] Acerbi I, Cassereau L, Dean I, Shi Q, Au A, Park C (2015). Human breast cancer invasion and aggression correlates with ECM stiffening and immune cell infiltration. Integr Biol.

[28] Winkler J, Abisoye-Ogunniyan A, Metcalf KJ, Werb Z (2020). Concepts of extracellular matrix remodelling in tumour progression and metastasis. Nat Commun.

[29] McConnell JC, O’Connell OV, Brennan K, Weiping L, Howe M, Joseph L (2016). Increased peri-ductal collagen micro-organization may contribute to raised mammographic density. Breast Cancer Res.

[30] Martin MD, Carter KJ, Jean-Philippe SR, Chang M, Mobashery S, Thiolloy S (2008). Effect of ablation or inhibition of stromal matrix metalloproteinase-9 on lung metastasis in a breast cancer model is dependent on genetic background. Cancer Res.

[31] Bui T, Rennhack J, Mok S, Ling C, Perez M, Roccamo J (2019). Functional redundancy between beta1 and beta3 integrin in activating the IR/Akt/mTORC1 signaling axis to promote ErbB2-driven breast cancer. Cell Rep.

[32] Smith HW, Hirukawa A, Sanguin-Gendreau V, Nandi I, Dufour CR, Zuo D (2019). An ErbB2/c-Src axis links bioenergetics with PRC2 translation to drive epigenetic reprogramming and mammary tumorigenesis. Nat Commun.

[33] Graus-Porta D, Blaess S, Senften M, Littlewood-Evans A, Damsky C, Huang Z (2001). Beta1-class integrins regulate the development of laminae and folia in the cerebral and cerebellar cortex. Neuron..

[34] Jonkers J, Meuwissen R, van der Gulden H, Peterse H, van der Valk M, Berns A (2001). Synergistic tumor suppressor activity of BRCA2 and p53 in a conditional mouse model for breast cancer. Nat Genet.

[35] Gunther EJ, Belka GK, Wertheim GB, Wang J, Hartman JL, Boxer RB (2002). A novel doxycycline-inducible system for the transgenic analysis of mammary gland biology. FASEB J.

