# Supplementary material for: Emergence of β1 integrin-deficient breast tumours from dormancy involves both inactivation of p53 and generation of a permissive tumour microenvironment
Source: Oncogene. 2021 Nov 15;41(4):527–37. doi: 10.1038/s41388-021-02107-7 (PMC8782722; doi:10.1038/s41388-021-02107-7)
Supplement: Supplementary file 1 — Supplementary information [file 41388_2021_2107_MOESM1_ESM.pdf]

Supplementary materials and methods – Bui et. al.

Mammary tissue collection. Mammary cancer tissues were collected at various time points throughout our study. Mammary tissues collected at 2 week-post Dox induction contain MIN lesions confirmed by H&E pathological evaluation. In this study, early invasive carcinoma refers to firm, palpable individual lesion at tumour onset that remains small (less than 5 mm by 5 mm in dimensions). Early invasive carcinomas were collected at variable times post-tumour onset due to different rates of tumour growth between lesions. In general, early invasive lesions were harvested within 3 weeks post palpation for MIC WT mice and within 8 weeks for MIC β1KO mice. When an individual tumour or the total tumour mass reaches the end-point burden defined by McGill Animal Ethics Guidelines, tumours collected at this stage are referred to as late invasive carcinoma or end-burden tumours. Pathological details of these lesions are described in Fig. S2a. Information regarding the types of mammary lesions in each analysis is provided in figure legends. For primary mammary tumour count at necropsy, individual distinct tumours of measurable size using a caliper are identified and counted. They can be of various size and stemming from any of the ten mammary glands.

Mammary gland whole mount staining. Number 4 mammary glands (2 week-post Dox induction) were fixed in acetone for at least 24 hours and subsequently stained with hematoxylin (Fisher) overnight. Tissues were then de-stained in 1% HCl (vol/vol) 70% ethanol, dehydrated in 100% ethanol and xylene overnight, and mounted with Permount (Fisher). Images were acquired using Axio Zoom V16 (Zeiss).

Lung metastasis scoring. Lungs were collected at end-point burden according to McGill Animal Ethics Guidelines. Tissues were fixed for 24 hours in 10% (vol/vol) formalin (Leica), embedded in paraffin and sectioned at 4 μm. Five step sections per lung were stained with H&E method by Histology Services at McGill University, scanned using Scanscope XT Digital Slide Scanner (Aperio Technologies) and analyzed using Aperio ImageScope software (Leica). Metastatic lung lesions were manually identified using H&E images. Quantification was done for total number and total area of lung lesions. Lesions appearing in multiple step sections were counted only once.

***In situ* cell detachment analysis.** To assess epithelial cell detachment from basement membrane, pathological examination was performed using H&E images of mammary gland 2 week-post Dox induction. At this stage, mammary glands are mostly MIN structures. Distances were measured for areas where epithelial layers are separated from the basement as well as for the total area of basement membrane (circumference) using Aperio ImageScope (Leica). Areas of cell detachment were presented as percentage of total circumference. At least 30 random MIN lesions were quantified per gland.

Immunohistochemistry (IHC), imaging and quantitative analysis. Primary tumours and lungs were harvested at end-burden. Tissues were fixed for 24 hours in 10% (vol/vol) formalin (Leica), embedded in paraffin, and sectioned at 4 μm. Tissues sections were stained with H&E by Histology Services at McGill University and scanned using Scanscope XT Digital Slide Scanner (Aperio Technologies). Antigen retrieval was performed by boiling samples in 10 mM citrate buffer (pH 6.0) or EDTA buffer (pH 9.0) (Vector Laboratories). Tissues were blocked for 10 min with Universal Blocking Agent (Biogenics) and further treated with 3% (vol/vol) H_2O_2 for 10 min.

Antibodies were prepared in 2% (wt/vol) BSA in PBS. For IHC, immunohistochemical labeling was performed using ImmPRESS polymers detection kit (Vector Laboratories) and DAB substrate kit (Cell Signaling Technologies). IHC images were acquired using an Aperio-XT Slide Scanner (Aperio Technologies). Image quantification was done using positive nuclear or positive pixel algorithm modules in Aperio ImageScope (Aperio Technologies). At least 10 random fields per tumour were quantified. For fluorescent IHC, following primary antibody, tissues were treated with ImmPRESS polymers detection kit (Vector Laboratories) and TSA reagents (Thermo Fisher). Immunofluorescence images were taken using an LSM800 Confocal Microscope (Carl Zeiss) or Axio Scan Z1 digital slide scanner (Carl Zeiss). Image quantification was done using HALO 2.0 platform (Indica Lab) and the algorithm 'Fluorescent intensity' or 'multiplex IHC v2.3.4'.

Shortest distances were measured using a built-in Imaris 9.02 (Bitplane) algorithm based on Euclidean shortest path method. Briefly, cells positive for each fluorescent channel were reduced to a point. Each tumour field at this point is a map consisting of 2 sets of points representing either Ki67+ cells or α-sma+ cells. A distance from each Ki67+ cell to a nearest neighbouring α-sma+ cell was measured. Overall, distances were measured for a total of at least 5000 cells per lesion type per genotype. Distribution curves of distances were generated using GraphPad Prism 5.

The following antibodies were used for IHC analyses: Ki67 (Cell Signaling Technology CST, 12202, 1:200), α-sma (Abcam, Ab7817, 1:200), cleaved caspase 3 (CST, 9661, 1:200), PyV mT (a generous gift from Dr. Stephen Dilworth, Ab750, 1:1,000), p16INK4a (Abcam, Ab54210, 1:200), p53 (Santa Cruz, sc-6243, 1:200), Rb (BD, 554136, 1:200), pRb S807/811 (CST, 8516, 1:200), cyclin D1 (CST, 2978, 1:200), Cre (CST, 12830, 1:200), CK8 (Fitzgerald, 20R-CP004, 1:200), β1 integrin (CST, 34971, 1:200), CK14 (Ventana, 760-4805, 1:10), and Desmin (CST, 5332, 1:100).

Isolation and culture of primary mammary cancer cells. Primary mammary cancer cells (MMTV-PyV mT) were obtained from mouse breast tumours. Tumours were processed with the McIlwain Tissue Chopper and disassociated in DMEM with 2.4 mg/mL Collagenase B (Roche) and 2.4 mg/mL Dispase II (Roche) for 2 hours rotating at 37°C. Dissociated cells were washed with ACK lysis buffer (150 mM NH_4Cl, 10 mM $KHCO_3$, 0.1 mM Na_2EDTA, pH 7.5) to remove red blood cells, then in 1 mM EDTA in PBS, passed through cell strainer, and plated on plastic. Cells were maintained in complete media (DMEM with EGF (5 ng/mL), bovine pituitary extract (35 μg/mL), insulin (5 μg/mL), hydrocortisone (1 μg/mL), penicillin (100 units/mL), streptomycin (100 μg/mL), gentamicin (50 μg/mL)) supplemented with 5% vol/vol FBS.

Cell adhesion assay. Primary epithelial cells were freshly isolated from 2 week-post Dox induction glands by method described above. 96 well plate was coated with FBS (2 hours at 37°C), Collagen I (Advanced Biomatrix, 1:60, overnight shaking at 4°C), or fibronectin (Sigma, 5 μg/mL, overnight shaking at 4°C), and rinsed with PBS before cell plating. 2 x 10^4 epithelial cells were plated in DMEM in quadruplicate. After 6 hours, non-adhered cells were removed by washing with PBS before processing for immunofluorescent staining. Briefly, cells were fixed in 2% PFA (20 min), permeabilized in 5% Triton-X (20 min), blocked in using Li-Cor Odyssey Blocking Buffer (30 min), and incubated in primary pan-Cytokeratin antibody (Cell Signaling Technology CST, 4545, 1:200, overnight at 4°C) to detect mammary epithelial cells and in secondary antibody (Li-Cor, 1:10,000, 1 hour at room temperature). Plate was scanned using Li-Cor Odyssey Scanner

to determine the level of cytokeratin. The staining signal intensity was quantified for each well, using Image Studio Lite software (Li-Cor).

3D tumour spheroid culture from primary cell lines. Established MMTV-PyV mT cell lines were first infected with ad5CMV adenoviral particles coding for AdLacZ, AdGFP, AdCre, AdCre-GFP or an empty vector (Vector Core, Center for Gene Therapy, University of Iowa). Adenoviruses were diluted in complete media (described above) with 2% vol/vol FBS. Infection was done at a multiplicity of infection (MOI) of 25. Cells were infected for 24 hours in a humidified 37°C incubator with 5% CO_2. Adenoviral particle-containing media was removed and replenished with complete DMEM supplemented with 5% vol/vol FBS for an additional 72 hours (MMTV-PyV mT cells lines show *Itgb1* ablation 96 hours post-infection), then trypsinized and treated with 1 mM EDTA in PBS. 10^4 cells were seeded on sterile coverslips coated with Geltrex and cultured in complete media with 5% vol/vol FBS for 6 to 8 days until tumour spheroids reached desirable size.

Edu incorporation assay. Edu incorporation was done using the Click-iT™ EdU Cell Proliferation Kit (Alexa Fluor™ 555 dye, Thermo Fisher) according to the manufacturer's protocol. For Edu incorporation, tumour spheroids were cultured in media containing Edu for 3 hours before fixation and processing for Edu detection. Following Edu detection, spheroids were then processed for immunofluorescent staining using the remaining available fluorescent channels. Images were acquired using LSM 800 confocal microscope (Leica) and analysis was performed using HALO 2.0 software (Indica Lab). At least 15 spheroids were analyzed per condition.

3D tumour spheroid immunofluorescence (IF) staining. Tumour spheroids (cultured as described above) were fixed in 2% paraformaldehyde (20 min at room temperature), permeabilized in 0.5% Triton-X (20 min at room temperature) and blocked in 2% BSA (30 min at room temperature). Spheroids were incubated in primary β1 integrin antibody (Sigma, Mab1997, 1:200 prepared in 2% BSA, overnight shaking at 4°C) and secondary Alexa Fluor conjugated antibodies (prepared in 2% BSA, 1 hour at room temperature). Nuclei were counterstained using Dapi. Images were acquired using LSM 800 confocal microscope (Leica) and analysis was performed using HALO 2.0 software (Indica Lab). At least 15 spheroids were analyzed per condition.

Senescence-associated β-Galactosidase assay. Fresh tissues (2 week-post Dox induction) were embedded in O.C.T. compound and flash-frozen in liquid nitrogen. Tissues were sectioned at 20 µm thickness using a cryotome and fixed in 2% paraformaldehyde in PBS for 20 min at room temperature. After washing twice with PBS, tissues were incubated in staining solution (0.1% X-Gal, 5 mM potassium ferrocyanide, 5 mM potassium ferricyanide, 150 mM sodium chloride, and 2 mM magnesium chloride in 40 mM citric acid/sodium phosphate solution, pH 6.0) overnight at 37°C (non-humid incubator). Tissues were rinsed in warm dH_2O to remove precipitated salt crystals before being counter-stained in hematoxylin. Stained sections were scanned using Scanscope XT Digital Slide Scanner (Aperio Technologies) and analyzed using ImageScope software. Only epithelial areas (MIN lesions) were selected for quantification (percentage of β-Galactosidase-positive cells).

For senescence-associated β-Galactosidase assay using 3D organoids, MMTV-PyV mT cells were infected with AdGFP or AdCre and cultured in geltrex on coverslips as described above. At day

10 post-infection, tumour spheres were fixed and stained using the conditions above. Stained tumour spheroids were mounted on glass microscope slides using Immu-Mount (Thermo Fisher). Bright field images were acquired by Axio Zoom V16 microscope (Zeiss). Each AdGFP and AdCre conditions are performed in triplicate. β-Galactosidase-positive spheres were scored manually. Spheres comprising a minimum of 30% β-Galactosidase activity-positive cells are considered β-Galactosidase-positive spheres.

Masson's trichrome staining and analysis. Tumour sections (4 μm) were stained for collagen fibers using Masson's trichrome, light green technique. Staining was performed by Goodman Cancer Institute Histology Core (McGill University). Images were acquired using Scanscope XT Digital Slide 156 Scanner (Aperio). Trichrome signal counts were quantified using a positive pixel module in ImageScope software (Aperio Technologies).

Immunoblot sample preparation and analysis. Four days after viral infection, MMTV-PyV mT cells were harvested for immunoblot analysis. Cells were collected in Eppendorf tubes, and washed once in PBS. Samples were incubated in complete lysis buffer (10 mM Tris-Cl pH 8.0, 1 mM EDTA, 0.5 mM EDTA, 1% Triton X-100, 0.1% sodium deoxycholate, 0.1% sodium dodecyl sulfate, 140 mM sodium chloride, 2 mM sodium pyrophosphate, 5 mM sodium fluoride, 10 mM β-glycerophosphate) with protease inhibitors (AEBSF 50 μg/mL, aprotinin 10 μg/mL, Leupeptin 10 μg/mL, Na_3VO_4 100 μg/mL). Samples were incubated in 500 μL of complete lysis buffer for 1 hour rotating at 4°C, centrifuged at maximum speed for 15 min, and supernatant was collected. The protein concentration in supernatant was determined from OD reading by diluting in Protein Assay Dye (Bio-Rad) and calculated in reference to a BSA standard curve. Loading samples were prepared by mixing supernatant to a final protein concentration of 2 μg/μL, 6X protein loading buffer (375 mM Tris-HCl, 10% SDS, 35% Glycerol, 0.012% bromophenol blue, 9.3% DTT, 5% β-mercaptoethanol), and complete lysis buffer. All samples were denatured at 95°C for 10 min and stored at -20°C. Equal quantity of protein per sample was loaded on acrylamide gel for running at 120 V then transferred onto Immobilon®-FL PVDF transfer membrane for 90 min at 24 V at 4°C. Membranes were locked using Li-Cor Odyssey® Blocking Buffer (TBS) for 1 hour at room temperature, incubated in primary antibodies (β1 integrin, BD 610468, 1:500; β-actin, Sigma, 1:2,000) overnight at 4°C, washed in TBS with 1% Triton X-100, incubated in secondary antibodies (1:10,000) for 1 hour at room temperature, washed, and imaged using Li-Cor Odyssey Scanner. Band intensity quantification was done using Image Studio Lite software (Li-Cor).

RNA extraction and RT-qPCR analysis. Total RNA was isolated using RNeasy kit (Qiagen). MMTV-PyV mT cells were first infected with AdGFP or AdCre-GFP and FACS sorted for successfully infected GFP+ cells 24 hours post-infection. GFP+ sorted cells were plated in complete media (described above) supplemented with 5% vol/vol FBS. Total RNA was extracted at day 4 post-infection, using RNeasy kit (Qiagen) and 20G needles. RNA quantity was determined using NanoDrop Spectrophotometer ND-1000 (NanoDrop Technologies, Inc.). cDNA was synthesized by reverse transcription using the TranScript all-in-one first strand cDNA synthesis kit (Transgen Biotech). Real-time qPCR was performed using LightCycler 480 SYBR Green I Master Reagents (Roche). Data were normalized to *Gapdh* to generate the relative transcript levels using the expression $2^{(\text{crossing point value of } Gapdh \, - \, \text{crossing point value of gene of interest})}$. Each reaction was run in triplicate. The following primers were used for RT-qPCR analysis: p16ink4a - left primer: GCGGACTCCATGCTGCTC, right primer: CACGACTGGGCGATTGGG; p19arf - left primer:

CGCAGGTTCTTGGTCACTGT, right primer: TGTTCACGAAAGCCAGAGCG; p53 (*Trp53*)- left primer: GTCGTACCCCGATTCAGGTG, right primer: TCTGCACCGTAGTTGAGCAG; p21 (*Cdkn1a*) - left primer: GGCAGACCAGCCTGACAGAT, right primer: TTCAGGGTTTTCTCTTGCAGAAG; *Gapdh* - left primer: CTGCACCACCAACTGCTTAG, right primer: GTCTTCTGGGTGGCAGTGAT.

Mouse p53 Sanger sequencing. DNA was extracted from frozen tumour pieces using phenol chloroform method. DNA samples were sent to Sanger sequencing service (primer design, sequencing reaction, and SNP analysis) at Génome Québec Innovation Center, McGill University. Results are summarized in Supplemental Table 1. The following primers were used to amplify the gene fragments: Trp53E01- left primer: GGAGAATCCTGACTCTGCAA, right Primer: CTTCCATTCCGCCCATCT; Trp53E02 - left primer: ACGTGGTTGGTTACCTCTGC, right Primer: GATACAGGTATGGCGGGATG; Trp53E03E04 - left primer: CCAGCCTGGGATAAGTGAGA, right Primer: GCTAAAAAGGTTCAGGGCAAA; Trp53E05E06 - left primer: TGGTGCTTGGACAATGTGTT, right Primer: CCCTTCTCCCAGAGACTGCT; Trp53E07E08E09 - left primer: GTAGGGAGCGACTTCACCTG, right Primer: AAGACCTGGCAACCTGCTAA; Trp53E010 - left primer: GTTGGGAACCAACTTTCAGA, right Primer: TGTCCCTCATACCCCTTAACA; Trp53E011 - left primer: CAGAAGTATTCCAGTGTGTTCTGTG, right Primer: CTACTCAGAGAGGGGGCTGA.

RNA-seq data acquisition and analysis. Library construction, quality assessment and sequencing (Illumina platform PE150) were performed by Novogene using pooled RNA samples from late invasive carcinomas (MIC WT n = 6, MIC β1KO n = 9 end-burden tumours). Data analyses including differentially expressed genes (DEGs) and GO enrichment were performed by Novogene according to their standard RNA-seq analysis pipeline. For heatmap and hierarchical clustering, DEGs with p values adjusted for multiple testing (FDR) <0.05 were clustered for similar expression using standardized FPKM values (Z-scores). Dendrograms were generated by hierarchical clustering on the Euclidean distances between genes (row) or samples (column) using Ward's method and Complete-linkage agglomeration (from the R package *hclust*), respectively. For ingenuity pathway analysis (IPA), 238 DEGs were analyzed to identify pathways being activated according to the software provider's instruction (Qiagen). Top 20 significant pathways with highest activation score were selected. For E2F1 gene signature, list of 238 DEGs was cross-referenced with a dataset, ENCODE transcription factor targets for E2F1 (Ma'ayan Laboratory of Computational System Biology, NCBI Gene Entrez Database 1869, http://www.ncbi.nlm.nih.gov/gene/1869) [1]. A heat map for E2F1 signature was created using overlapped genes between the two gene lists. Raw and processed data are available on GEO repository under accession number GSE186491.

Single cell RNA-seq sample acquisition and analysis. Early invasive carcinoma lesions (described above) were taken and removed from excess fat and surrounding tissues. A small piece of tumour was taken for DNA extraction and PCR for genotype confirmation (described above). Tumours were dissociated into single cell suspension following the "Isolation and culture of primary mammary cancer cell" protocol. Cells were blocked with TruStain FcX™ (BioLegend, 1:300) and stained with Viability Dye eFluor506 (Thermo Fisher, 1:1000) in FACS buffer (2% vol/vol FBS and 2 mM EDTA in PBS). Cells were sorted at the Flow Cytometry Innovation

Platform at McGill University and negatively stained viable cells were collected back in PBS. Library construction (10X genomics single cell 3' transcriptome library), quality assessment and sequencing (Illumina HiSeq 4000) were performed by Génome Québec Innovation Tumour. All scRNA-seq analyses were performed by the Canadian Tumour for Computational Genomics (C3G). Using Cell Ranger's *count*, reads were first aligned to a custom mm10 mouse reference genome that included Cre recombinase and PyV mT. The resulting UMI count matrices (genes × cells) were then provided as input to Seurat suite (version 3.2.3) [2, 3]. A minimum of 800 and a maximum of 8000 detected genes per cell was defined as acceptable range to exclude low-quality cells. Cells with more than 8% of the transcript counts derived from mitochondrial-encoded genes were further removed. Single cell RNA Seq datasets were integrated using Seurat's alignment procedure. Briefly, canonical correlation analysis (CCA) was performed to identify shared sources of variation to produce anchors across the datasets following *SCTransform* normalization. Clustering and visualization of the integrated dataset were performed using Uniform Manifold Approximation and Projection (UMAP), based on the first 11 principal components with a resolution of 0.3 (*FindClusters* and *RunUMAP* functions in Seurat). Cluster-specific marker genes were identified using *FindAllMarkers* function with a cut-off of log fold-change > 0.5 and Bonferroni adjusted p value < 0.05, and cell-type annotation was performed using SCSA [4]. Differential gene expression analysis between β1 integrin-proficient and -deficient cells was performed using *FindMarkers*. Differentially expressed genes were identified using a cut-off of |log fold-change| > 0.25 and Bonferroni adjusted p value < 0.05. Raw and processed data are available on GEO repository under accession number GSE186118.

Statistical analysis. All statistical analyses were done using GraphPad Prism 5 software. Significance between 2 sets of data was assessed using two-tailed Students' t-test or 2-way ANOVA test as indicated in figure legends. Data represent mean ± SEM (standard error of the mean) for biological replication or ± SD (standard deviation) for technical replication. For Kaplan Meier survival analysis, statistical significance was calculated by Lox-rank Mantel Cox test. P values < 0.05 are considered significant.

References

1. Rouillard, A.D. *et al.* The harmonizome: a collection of processed datasets gathered to serve and mine knowledge about genes and proteins. *Database (Oxford)* **2016** (2016).
2. Butler, A., Hoffman, P., Smibert, P., Papalexi, E. & Satija, R. Integrating single-cell transcriptomic data across different conditions, technologies, and species. *Nat Biotechnol* **36**, 411-420 (2018).
3. Satija, R., Farrell, J.A., Gennert, D., Schier, A.F. & Regev, A. Spatial reconstruction of single-cell gene expression data. *Nat Biotechnol* **33**, 495-502 (2015).
4. Cao, Y., Wang, X. & Peng, G. SCSA: A Cell Type Annotation Tool for Single-Cell RNA-seq Data. *Front Genet* **11**, 490 (2020).

Supplemental figure S1 (Bui et. al.)

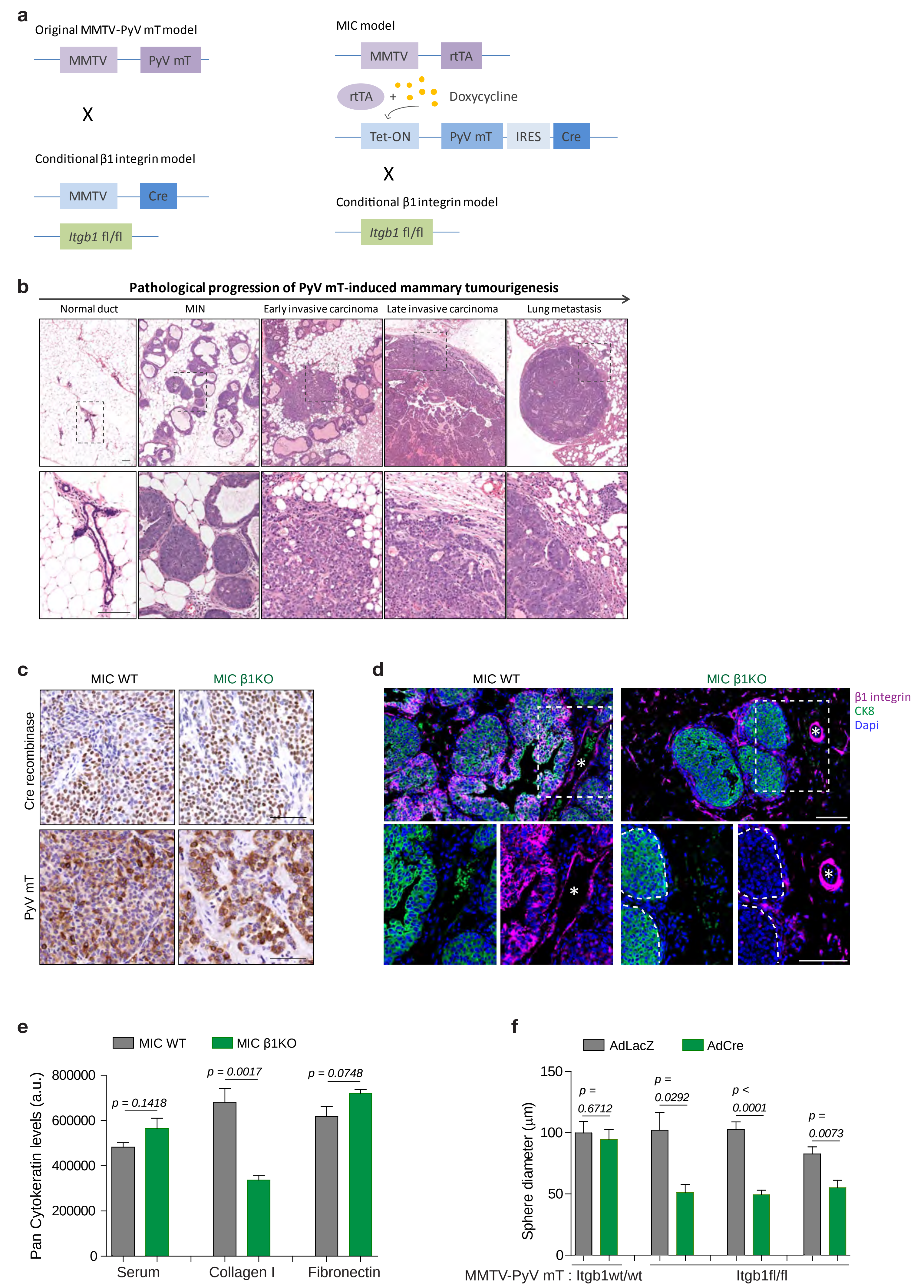

Figure S1 – Mouse models of PyV mT-driven mammary tumourigenesis.

a Schematic representation of PyV mT-driven mouse models. Original MMTV-PyV mT model relies on MMTV-Cre to generate β1 integrin knockout mice. Stochastic expression of the two MMTV promoters allows ‘escapee’ tumours that still retain β1 integrin expression due to β1 integrin-dependent growth advantage. Recent MIC model employs IRES element to couple PyV mT and Cre recombinase expression, thereby preventing ‘escapee’ phenomenon.

b H&E images of stepwise progression of PyV mT-induced mammary tumourigenesis. Pathological progression of MIC mice highly resembles pathological stages observed in human breast cancer. Scale bars are 100 µm.

c Immunohistochemical analyses confirm expression of Cre recombinase and PyV mT oncogene in mammary tumours (late invasive carcinoma) of the indicated genotypes. Scale bars are 100 µm.

d Immunohistochemical analyses show epithelial-specific loss of β1 integrin expression in MIN (2 week-post Dox induction). CK8 labels mammary epithelial tumour cells lacking β1 integrin signal in MIC β1KO lesions. Asterisks indicate blood vessels that still display β1 integrin expression. Scale bars are 100 µm.

e Epithelial cells were freshly derived from MIC WT or MIC β1KO mammary glands 2 week-post Dox induction and plated on various substrates for 4 hours. Cells were then stained for pan-cytokeratin marker. Quantification of pan-cytokeratin was quantified and averaged from quadruplicate wells. The error bars indicate ±SD, two-tailed Student’s t-test.

f Average tumour sphere diameters from images in Fig. 3b. The error bars indicate ±SD, two-tailed Student’s t-test.

Supplemental figure S2

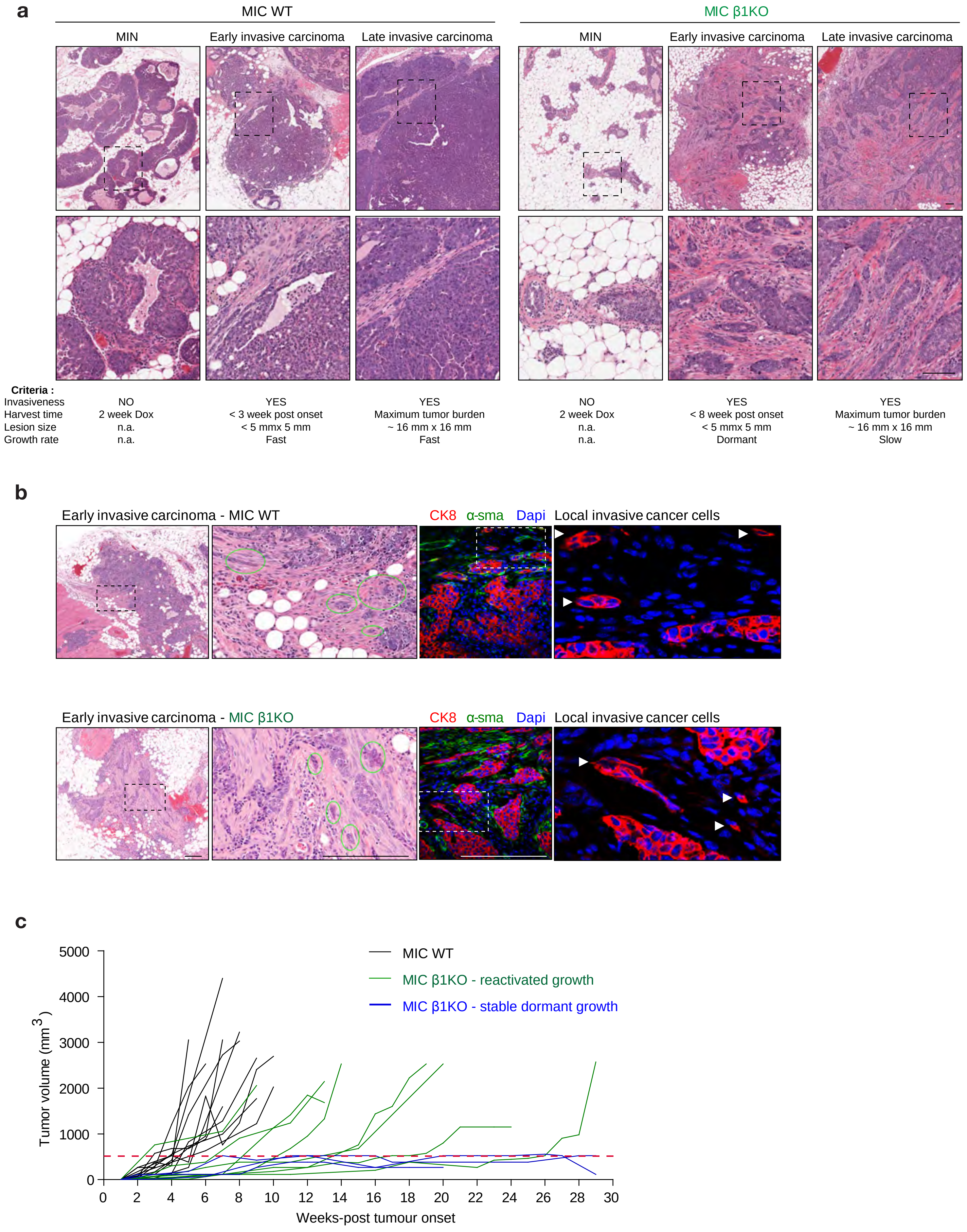

Figure S2 – Pathological progression of mammary tumourigenesis in the MIC model.

a Tissues analyzed in this study are classified into different stages, based on several criteria such as evidence of local invasion, size and time. H&E images illustrate representative pathology of those lesions. Scale bars are 100 µm.

b H&E images of early invasive carcinoma, which represents palpable tumours with small volume. This stage represents invasive carcinoma where invasive cells are outlined in green. Immunohistochemical analysis of CK8+ cancer cells, arrow heads indicate invasive cancer cells, either as single cell or collective groups. Scale bars are 100 µm.

c Tumour volumes were calculated for each individual mouse for the indicated genotypes. There are two distinct groups of MIC β1KO tumours, one with initial dormant growth and subsequent reactivated tumour growth (green) and one with stable dormancy (blue).

Supplemental figure S3

a

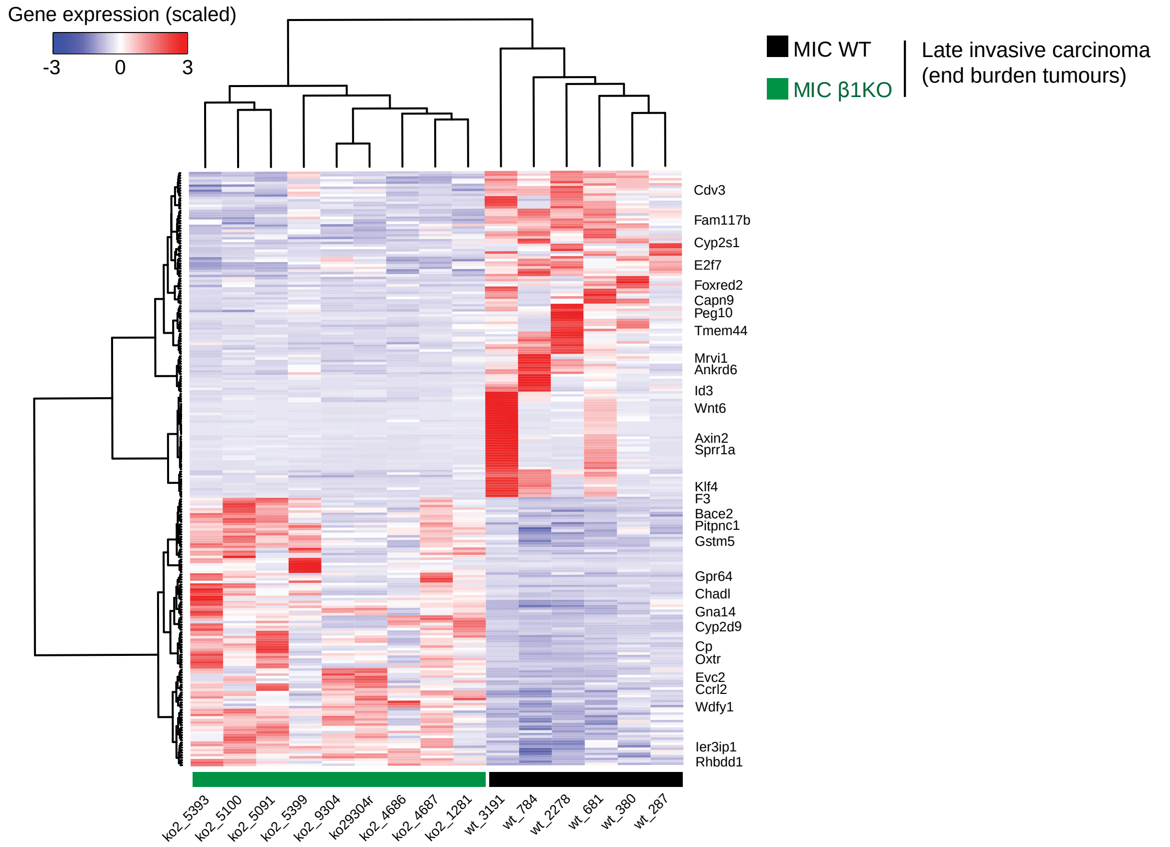

b

Upstream Regulator	Molecule Type	Predicted Activation State	Activation z-score	p-value of overlap
ERBB2	kinase	Inhibited	-2.557	3.42E-01
WNT3A	cytokine	Inhibited	-2.424	1.19E-05
MYC	transcription regulator	Inhibited	-2.217	7.67E-03
E2F1	transcription regulator	Inhibited	-2.198	2.25E-01
MET	kinase	Inhibited	-2	2.32E-02
EDN1	cytokine	Inhibited	-1.985	1.37E-01
lithium chloride	chemical drug	Inhibited	-1.972	2.69E-02
PPARG	ligand-dependent nuclear receptor	Inhibited	-1.964	5.15E-01
simvastatin	chemical drug	Inhibited	-1.963	2.05E-02
SP3	transcription regulator	Inhibited	-1.959	6.62E-02
Vegf	group	Inhibited	-1.929	3.18E-02
FOXM1	transcription regulator	Inhibited	-1.901	4.47E-02
valproic acid	chemical drug	Inhibited	-1.89	1.71E-01
KLF4	transcription regulator	Inhibited	-1.713	1.48E-02
GLI1	transcription regulator	Inhibited	-1.684	4.42E-03
THRB	ligand-dependent nuclear receptor	Inhibited	-1.664	5.44E-03
SRF	transcription regulator	Inhibited	-1.633	2.42E-03
TP63	transcription regulator	Inhibited	-1.626	4.03E-02
STAT3	transcription regulator	Inhibited	-1.536	3.00E-02
TNF	cytokine	Inhibited	-1.533	3.10E-02
SYVN1	transporter	Activated	1	4.78E-02
cyclophosphamide	chemical drug	Activated	1	6.47E-03
enalapril	chemical drug	Activated	1	1.81E-03
PDX1	transcription regulator	Activated	1.067	3.15E-02
captopril	chemical drug	Activated	1.067	9.89E-04
TP53	transcription regulator	Activated	1.183	5.06E-03
FOS	transcription regulator	Activated	1.195	1.29E-04
CEBPB	transcription regulator	Activated	1.29	3.16E-04
S100A9	other	Activated	1.342	2.76E-02
TREM1	transmembrane receptor	Activated	1.387	4.99E-02
SMARCA4	transcription regulator	Activated	1.648	3.54E-03
nitrofurantoin	chemical drug	Activated	1.664	1.44E-03
APC	enzyme	Activated	1.9	3.78E-05
pyrrolidine dithiocarbamate	chemical reagent	Activated	1.951	2.19E-02
GATA2	transcription regulator	Activated	1.982	3.67E-01
miR-199a-5p	mature microrna	Activated	2	5.32E-03
LEP	growth factor	Activated	2.045	1.38E-02
DKK1	growth factor	Activated	2.213	3.33E-04
CBX5	transcription regulator	Activated	2.236	6.46E-03
dexamethasone	chemical drug	Activated	3.471	3.88E-02

Figure S3 – RNA-seq analysis reveals major transcriptomic changes in β1 integrin-deficient tumours.

a Heat map representation of 238 differentially expressed genes (107 upregulated genes and 121 downregulated genes) in MIC β1KO tumours in comparison to MIC WT tumours.

b The list of 238 differentially expressed genes was analyzed by Ingenuity Pathway analysis (IPA) to identify activated or inhibited pathways in MIC β1KO tumours.

Supplemental figure S4

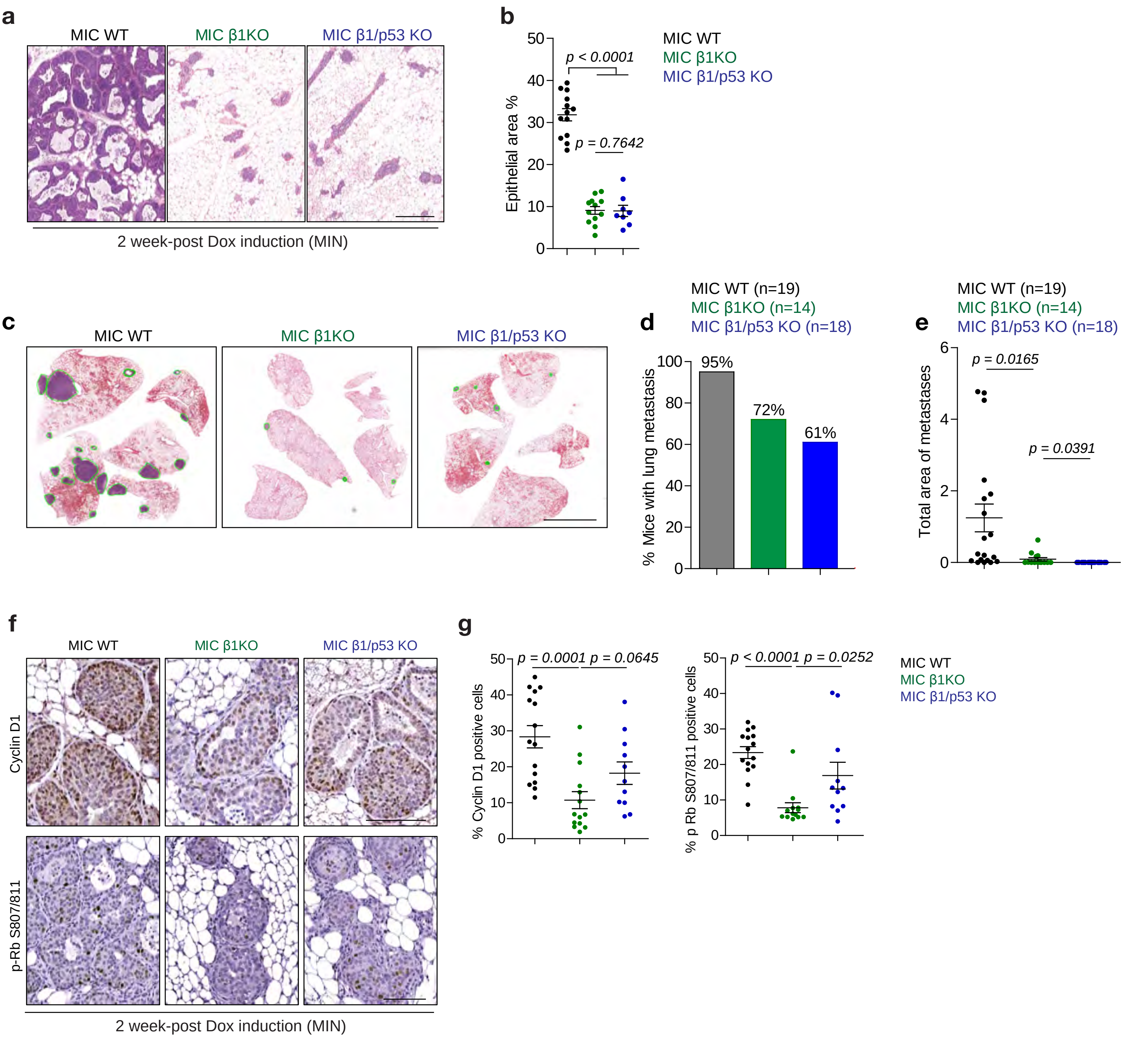

Figure S4 – p53 inactivation was unable to rescue tumour initiation and metastatic defects due to β1 integrin deficiency.

a Representative H&E images of mammary glands at 2 week-post Dox induction. Scale bar is 100 µm.

b Percentage of epithelial area, normalized to total gland area, from H&E images in (a).

c Representative H&E images of lung collected at end-burden. Green outlines indicate metastases. Scale bar is 5 mm.

d Lungs were collected when total tumour mass reached end-burden and analyzed for visible metastases using H&E images in (c). Bar graph shows percentages of mice with lung metastasis per genotype.

e Total area of metastatic lung lesions per animal. P values were calculated between MIC β1KO mice to each of the other cohorts.

f Immunohistochemical analyses of 2 week-post Dox induction MIN structures for cyclin D1 and phosphorylated Rb levels. Scale bars are 100 µm.

g Quantification of positive nuclei for cyclin D1 and phosphorylated Rb.

For all data, the center line indicates the mean and error bars indicate ±SEM, two-tailed Student’s t-test.

Supplemental figure S5

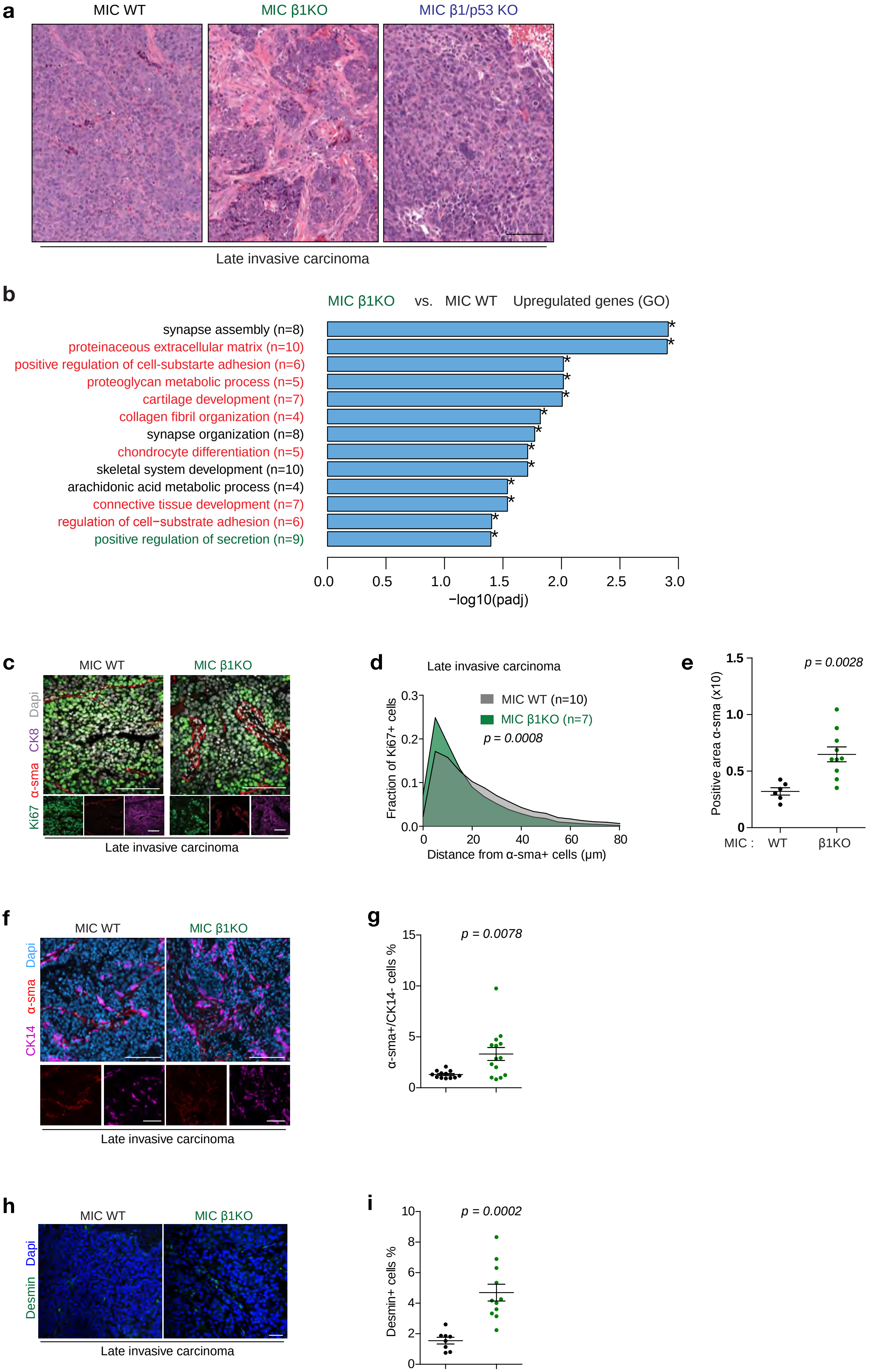

Figure S5 – Dormant β1 integrin-deficient tumours exhibit aberrant tissue fibrosis.

a Representative H&E images of tumours collected at end-burden (late invasive carcinoma). Stained cancer cells appear blue/purple whereas ECM appears pink. Scale bar is 100 µm.

b RNA-seq data from whole tumour bulk RNA pool (late invasive carcinoma). 107 genes upregulated in MIC β1KO tumours were subjected to GO term analysis to identify cellular processes being altered between MIC WT and MIC β1KO tumours. Horizontal axis indicates adjacent p values.

c Immunohistochemical analyses of late invasive carcinoma for Ki67, α-sma (CAF marker) and CK8 (mammary tumour cell marker). Scale bars are 100 µm.

d Analysis of the distribution of distances from a proliferative cancer cell (Ki67+/CK8+) to the nearest CAF (α-sma+) from images in (c) (MIC WT n = 10, MIC β1KO n = 7). P value is calculated using averaged distance for each lesion. Student’s t-test.

e Quantification of α-sma-positive areas from Fig. S5c images. Data ± SEM, two-tailed Student’s t-test.

f Representative images shows that majority of α-sma-positive cells are CK14-negative in late invasive tumours. Scale bar 100   m.

g Quantification of α-sma+/CK14- cells from Fig. S5f images. Data ± SEM, two-tailed Student’s t-test.

h Representative IHC images showing a CAF marker, Desmin. Scale bar 40 µm.

i Quantification of Desmin+ cell percentage from Fig. S5h images. Data ± SEM, two-tailed Student’s t-test.

Supplemental figure S6

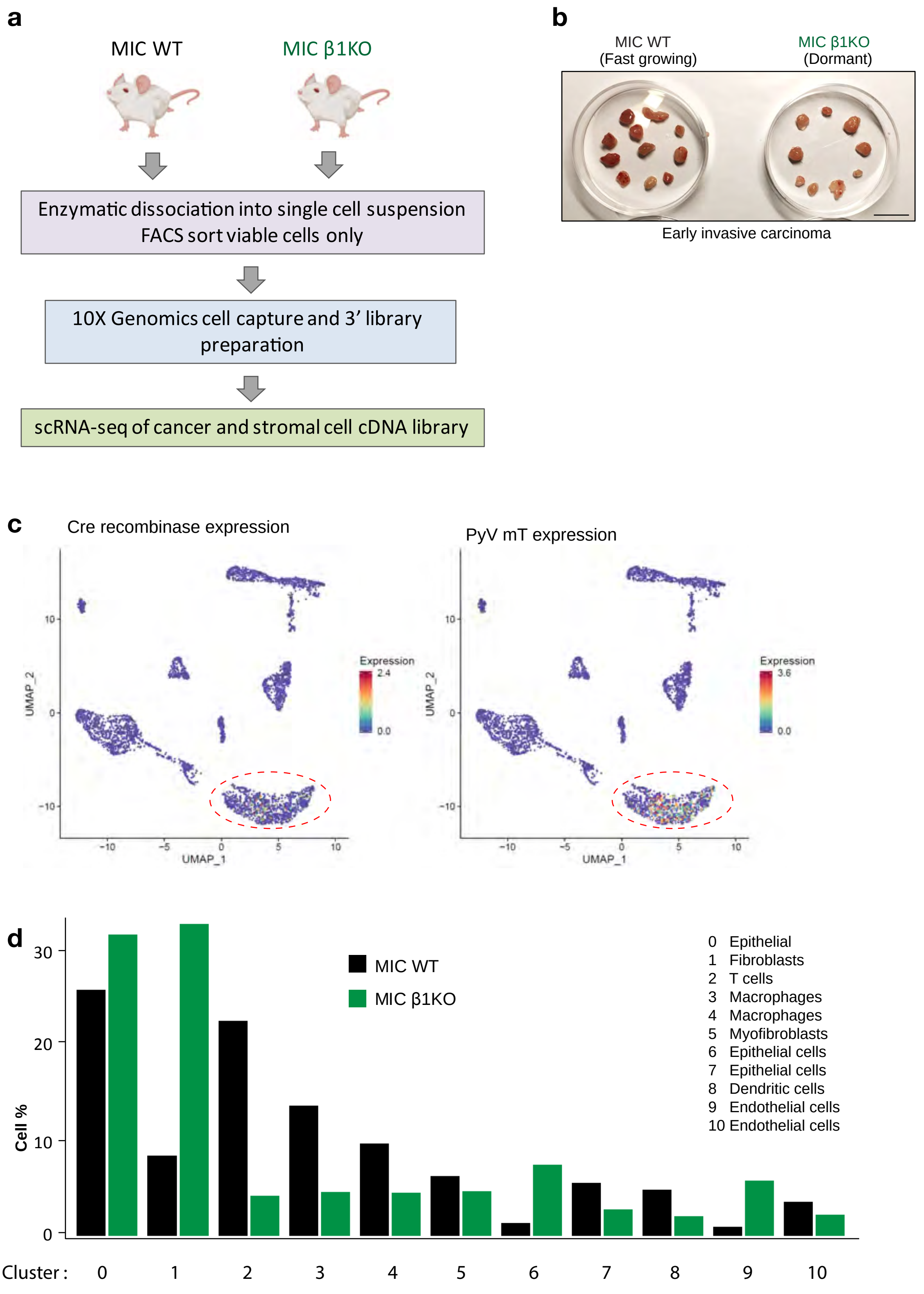

Figure S6 – Single cell RNA sequencing of dormant β1 integrin-deficient tumours.

a Schematic of sample preparation for scRNA-seq. Briefly, early invasive carcinomas representing two categories of lesions (fast growing MIC WT lesions and dormant MIC β1KO lesions) were harvested for scRNA-seq. RNA from only viable cells (both cancer and stromal cells) were used for library construction and sequencing.

b Images of lesions included in scRNA-seq. Lesions were pooled from 3 MIC WT mice and 6 MIC β1KO mice. Scale bar is 1 cm.

c Cre recombinase and PyV mT expression for all cells sequenced (pooled from both MIC WT and MIC β1KO lesions). Cluster 0 (outlined in red) displays the most cells positive for Cre/PyV mT expression and is therefore the main transgenic cancer cell cluster.

d Percentages of cell clusters identified using UMAP analysis between MIC WT (fast growing) and MIC β1KO (dormant) early invasive lesions.

Supplemental figure S7

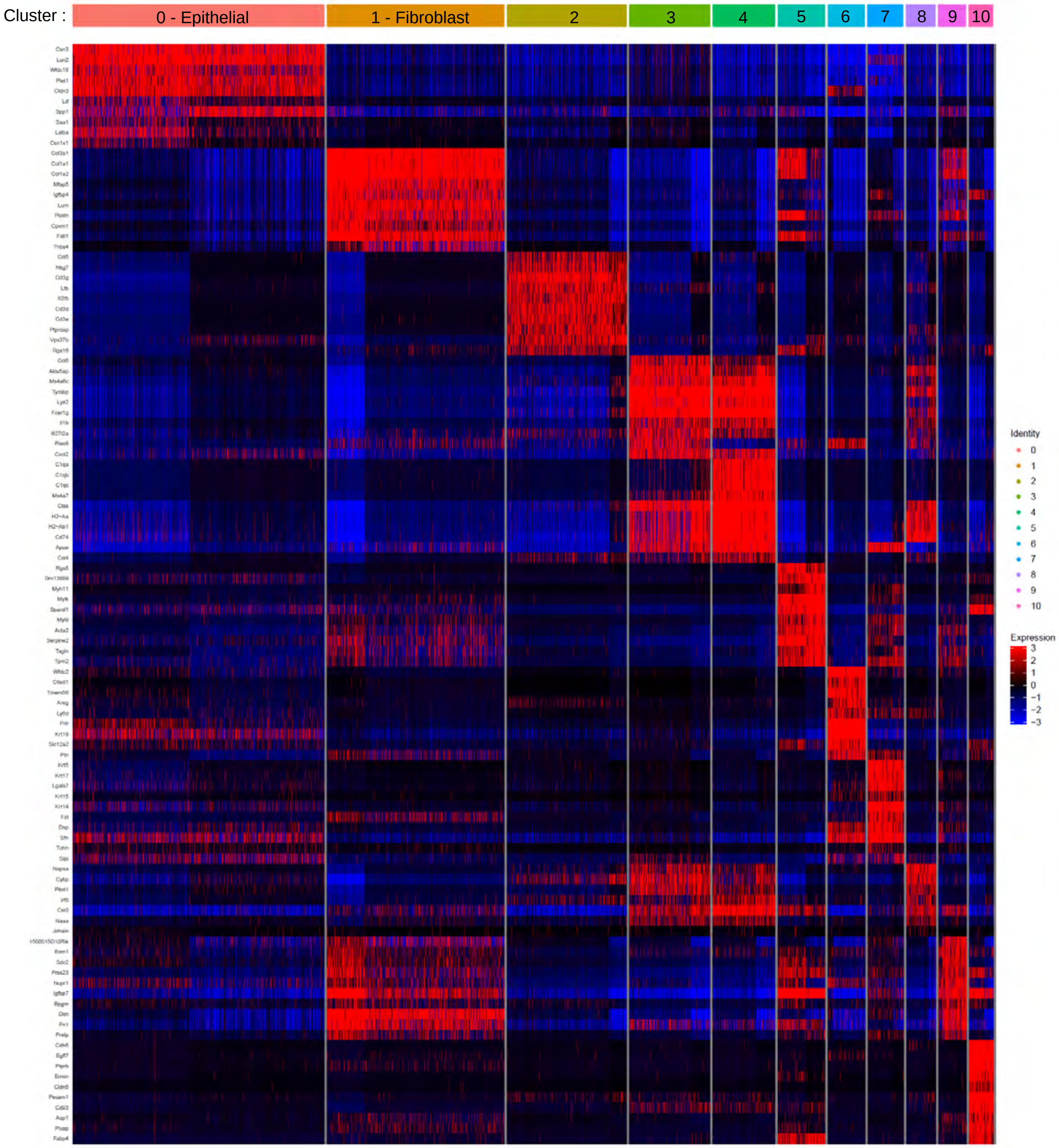

- 0 Epithelial
- 1 Fibroblasts
- 2 T cells
- 3 Macrophages
- 4 Macrophages
- 5 Myofibroblasts
- 6 Epithelial cells
- 7 Epithelial cells
- 8 Dendritic cells
- 9 Endothelial cells
- 10 Endothelial cells

Figure S7 – Cell cluster identify by single cell RNA-seq.

Heatmap showing top 10 expressed genes that were used to distinguish 11 cell clusters identified in UMAP analysis in Fig. 6c.
