# Supplementary material for: Emergence of β1 integrin-deficient breast tumours from dormancy involves both inactivation of p53 and generation of a permissive tumour microenvironment
Source: Oncogene. 2021 Nov 15;41(4):527–37. doi: 10.1038/s41388-021-02107-7 (PMC8782722; doi:10.1038/s41388-021-02107-7)
Supplement: Supplementary file 2 — Supplementary table 1 [file 41388_2021_2107_MOESM2_ESM.pdf]

Supplementary table 1 – Results of p53 Sanger sequencing for β1 integrin-deficient tumours

		Trp53E01	Trp53E02	Trp53E03E04
PCR fragment name		**Trp53E01**	**Trp53E02**	**Trp53E03E04**
Primers used		Trp53E01F	Trp53E02F	Trp53E03E04F
PCR size (*bp*)		426	344	585
Sequencing orientation		F	F	F
SNP success rate (%) ^_^		100.00%	100.00%	100.00%
SNP position in contig		360		424
SNP ID *(to be located in the text sequence)*		n/a		n/a
Genotype (*Wildtype>Mutant*)		G>C	--	Deletion GGGCCCC
Position in UCSC Genome Browser		chr11:69,580,621		chr11:69,587,639
Position in Intron/Exon/UTR/Promoter		Intron		Intron
Function (*Wiltype vs Mutant*)				
UCSC SNP reference number (Feb 2009)		n/a		n/a
**Major Allele / minor Allele ^_^**		**G (85.7%) / C (14.3%)**	**--**	**Normal (85.7%) / Deletion (14.3%)**
**Homozygous M / Homozygous m / Heterozygous ^_^**		**6 (85.7%) / 1 (14.3%) / 0 (0.0%)**	**--**	**Normal (85.7%) / Delhomo (14.3%) / Delhet (0.0%)**
**Sample names**	2946	GG	--	//
	3438	GG	--	//
	3549	GG	--	//
	3571	GG	--	//
	3610	CC	--	Delhomo_GGGCCCC
	4030	GG	--	//
	5740	GG	--	//
	**Surrounding Sequence**	gggtttgggggtggggcagtgggggGactcagcgcgatggagatgg	No SNP	agccaagtctgttatgtgcacggtgagtgggccccgggccccggggagttgtctttcgtgtgacc
	**Comments**	Done	Done	Redo 3438

PCR fragment name		Trp53E05E06	Trp53E07E08E09	Trp53E10	Trp53E11
Primers used		Trp53E05E06F	Trp53E07E08E09F	Trp53E10F	Trp53E11F
PCR size (*bp*)		645	864	370	390
Sequencing orientation		R	R	F	R
SNP success rate (%) ^_^		100.00%	100.00%	100.00%	100.00%
SNP position in contig		166			
SNP ID *(to be located in the text sequence)*		n/a			
Genotype (*Wildtype>Mutant*)		G>A	--	--	--
Position in UCSC Genome Browser		chr11:69,588,712			
Position in Intron/Exon/UTR/Promoter		Exon			
Function (*Wiltype vs Mutant*)					
UCSC SNP reference number (Feb 2009)		n/a			
**Major Allele / minor Allele ^_^**		**G (92.9%) / A (7.1%)**	**--**	**--**	**--**
**Homozygous M / Homozygous m / Heterozygous ^_^**		**6 (85.7%) / 0 (0.0%) / 1 (14.3%)**	**--**	**--**	**--**
**Sample names**	2946	GG	--	--	--
	3438	GG	--	--	--
	3549	GG	--	--	--
	3571	GG	--	--	--
	3610	GG	--	--	--
	4030	GG	--	--	--
	5740	GA	--	--	--
	**Surrounding Sequence**	ctggaagacaggcagacttttcgccacagcGtggtggtaccttatgagccacccgaggtc	No SNP	No SNP	No SNP
	**Comments**	Done	Done	Redo 2946	Done
